# Possible Applications of Cold Atmospheric Plasma in Otorhinolaryngology: A Narrative Review

**DOI:** 10.3390/antiox15081026

**Published:** 2026-08-17

**Authors:** José Andrés González Coraspe, Ramón Moreno-Luna, Fabian Görries, Maria Dib, Chia-Jung Busch, Christian Scharf

**Affiliations:** 1 Department of Otorhinolaryngology, Head and Neck Surgery, University Medicine Greifswald, 17475 Greifswald, Germany; jandresgonzalezc@gmail.com (J.A.G.C.); fabian.goerries@web.de (F.G.); maria.dib@med.uni-greifswald.de (M.D.); chia-jung.busch@med.uni-greifswald.de (C.-J.B.); 2 Rhinology and Anterior Skull Base Department, Hospital Universitario Virgen Macarena, 41009 Sevilla, Spain; ramoluorl@gmail.com; 3 Hospital Robert Koch Gehrden, 30989 Gehrden, Germany

**Keywords:** cold atmospheric plasma, otorhinolaryngology, wound healing, pathogen eradication, antitumor strategies, adjuvant tumor therapy, immunomodulation, CAP in ORL, PLASMASKOP

## Abstract

Cold atmospheric plasma (CAP), a non-thermal ionized gas, is gaining attention in biomedicine due to its unique properties such as the generation of reactive oxygen and nitrogen species (RONS), immunomodulatory effects, and selective cytotoxicity. This review explores the potential of CAP as a therapeutic tool in otorhinolaryngology (ORL). CAP has shown promising applications in wound healing, pathogen eradication, and antitumor strategies, particularly in head and neck cancers. Mechanistically, CAP promotes tissue regeneration through redox signaling, stimulates immune cell activity, induces immunogenic cell death, and enhances epithelial proliferation while offering broad-spectrum antimicrobial efficacy. The review also discusses the development of miniaturized CAP delivery systems, including endoscope-integrated plasma jets (e.g., PLASMASKOP), to facilitate clinical use in anatomically complex ORL regions. Given the nascent stage of clinical translation in ORL, the authors highlight the need for interdisciplinary research and device innovation to realize the full therapeutic potential of CAP in this field.

## 1. Introduction

In recent years, atmospheric-pressure plasma sources have attracted considerable attention in biomedicine owing to their versatility and expanding range of clinical applications. Plasma, frequently described as the fourth state of matter alongside solids, liquids, and gases, is a partially or fully ionized gas [1]. In the medical literature, the non-thermal form is referred to interchangeably as cold atmospheric plasma (CAP), low-temperature plasma (LTP), tissue-tolerable plasma (TTP), or non-invasive physical plasma (NIPP); for consistency, we use the term CAP throughout this review.

Two broad categories of plasma are distinguished. Thermal plasmas, such as argon or helium coagulation plasma, reach temperatures of up to several thousand degrees Celsius and are exploited in electrosurgery for their hemostatic and tissue-ablative effects [2,3]. In contrast, CAP is only partially ionized and is maintained at or near body temperature, typically between 37 °C and 42 °C, which allows it to be applied directly to living tissue without causing thermal damage [4]. Its biological activity arises not from heat but from the interaction of ionized particles, electric fields, UV photons, and—most importantly—reactive species with cells and tissues.

When the plasma effluent meets ambient air and the aqueous layer covering cells, it generates a spectrum of reactive oxygen and nitrogen species (RONS), including hydrogen peroxide, hydroxyl radicals, singlet oxygen, superoxide, nitric oxide, nitrogen dioxide, and peroxynitrite [5]. These RONS are now widely accepted as the principal mediators of CAP’s effects on biological systems, and their composition can be tuned through the feed gas, applied voltage, and treatment medium [6]. The intracellular and extracellular consequences of this reactive chemistry are considered in detail in Section 2.

Over little more than a decade, this reactive chemistry has been translated into a growing set of clinical and preclinical applications, establishing CAP as a genuinely cross-disciplinary technology. The most mature field is dermatology and chronic-wound care: PlasmaDerm^®^ and kINPen^®^ MED were certified as class IIa medical devices in Germany in 2013 for the treatment of chronic wounds and pathogen-related skin disease [7], and randomized controlled trials have since demonstrated improved healing in diabetic foot ulcers and chronic venous leg ulcers [8,9]. In parallel, the strong antimicrobial activity of CAP has been applied to the disinfection of medical equipment and even to the decontamination of flexible endoscopes, with efficacy against biofilms and multidrug-resistant organisms [10,11,12]. Related applications have emerged in dentistry [13], and CAP has additionally shown benefit in inflammatory skin conditions such as psoriasis [14]. Most recently, its selective cytotoxicity towards malignant cells has driven interest in oncology, culminating in the first FDA-approved Phase I trial of intraoperative CAP in advanced solid tumors [15]. CAP has been approved for medical use in Germany, the United States, and Japan, and reported to be safe and effective across multiple indications [16].

Despite this breadth of application, the translation of CAP into otorhinolaryngology (ORL) remains at an early stage, even though many of the disorders encountered in this field—chronic mucosal infections, impaired wound healing, biofilm-associated rhinosinusitis, and head and neck malignancy—align closely with the areas in which CAP has already proven useful. The principal obstacle is anatomical: the relevant target sites lie within narrow, difficult-to-access cavities of the nose, paranasal sinuses, pharynx, larynx, and ear, for which conventional CAP applicators are poorly suited. This has motivated the development of miniaturized and endoscope-integrated plasma sources, such as the PLASMASKOP discussed in Section 13 and Section 15. Within this context, the potential of CAP in ORL can be grouped into three principal domains—the promotion of wound healing, the eradication of pathogens (including the modulation of inflammatory conditions), and the treatment of cancer—together with their underlying mechanisms of action. This review brings together and critically appraises the currently available evidence across these domains, with the aim of providing an overview of the possible applications of CAP in ORL and a foundation for future research in this emerging field.

## 2. Intracellular and Extracellular Effects of CAP

**Figure 1:**
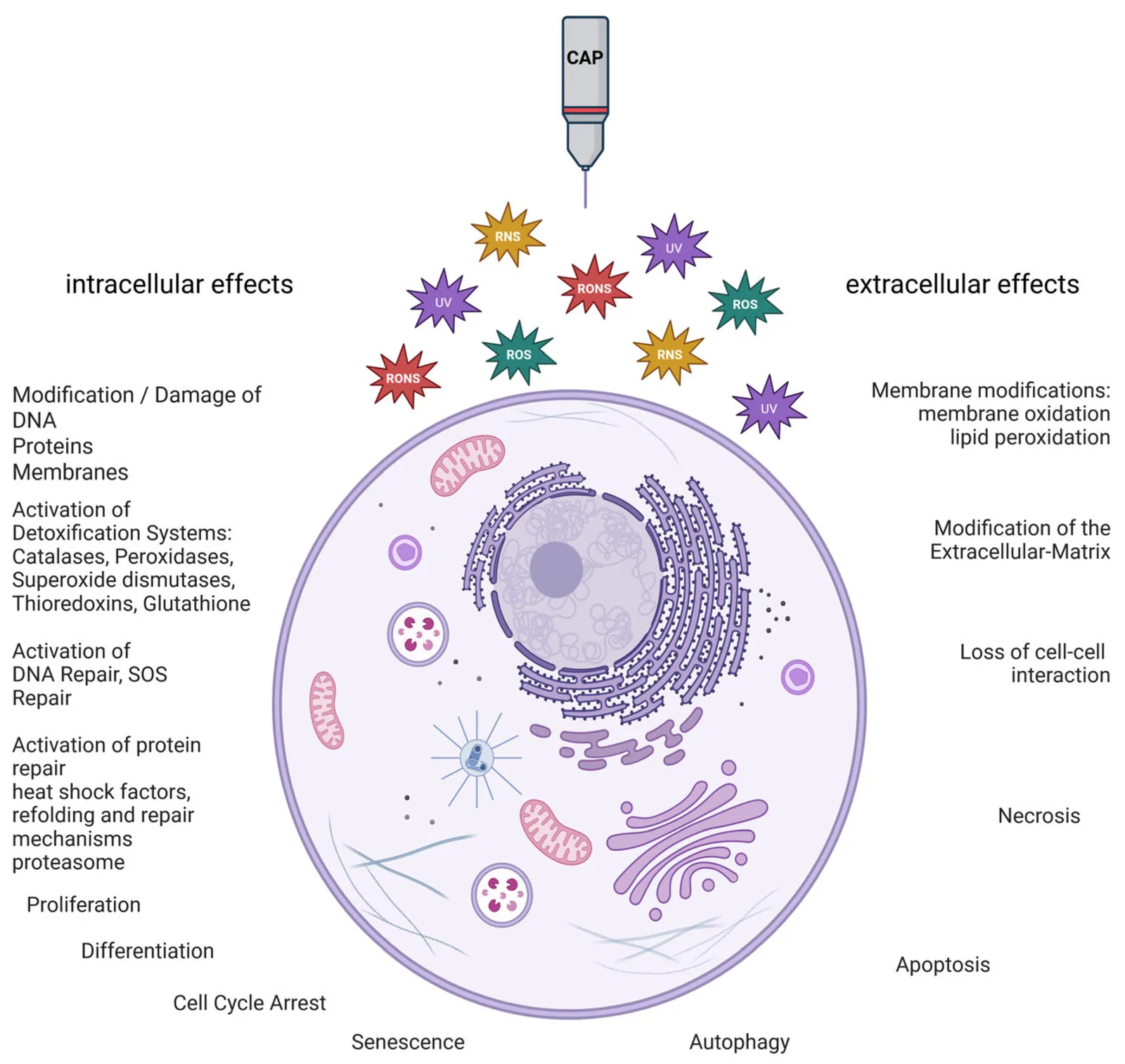
Effects of cold atmospheric plasma (created in BioRender. Scharf, C. (2026) https://BioRender.com/47c5wgv).

Understanding the mechanisms underlying the effects of CAP is essential both to maximize its therapeutic benefit and to define safe, effective treatment windows. CAP exerts its biological activity predominantly through the reactive oxygen and nitrogen species (RONS) generated when the plasma effluent interacts with ambient air and with the aqueous layer surrounding cells and tissues [6,17]. These species act at several levels—the plasma membrane and extracellular matrix, the cytosol and its antioxidant machinery, the mitochondria, and the nucleus—and the balance between adaptive survival responses and regulated cell death is strongly dose-dependent (Figure 1).

### 2.1. Cellular Uptake of Reactive Species

Before eliciting intracellular effects, RONS must either cross the plasma membrane or trigger signaling through modification of transmembrane proteins. The route of entry depends on the physicochemical nature of each species. Hydrophilic species such as hydrogen peroxide (H_2_O_2_) traverse the membrane largely through aquaporin channels [18], whereas lipophilic species such as nitric oxide and nitrogen dioxide can diffuse directly across the lipid bilayer [19]. This distinction is therapeutically relevant: many tumor cells overexpress aquaporins, which increases their H_2_O_2_ uptake and has been proposed as one basis for the preferential sensitivity of malignant cells to CAP [20].

### 2.2. Antioxidant Defense Systems and Redox Homeostasis

Eukaryotic cells counter oxidative challenge through several interconnected antioxidant systems, principally the thioredoxin and glutathione systems together with the peroxiredoxins [21]. The thioredoxin system reduces protein disulfide bonds to maintain the intracellular redox balance, and thioredoxin reductase 1 additionally regulates the Keap1–Nrf2 axis [22]. Glutathione (GSH) directly neutralizes reactive species and serves as the cofactor for glutathione peroxidases [23]. Peroxiredoxins, in particular the fast-reacting peroxiredoxin 2, provide a further layer of H_2_O_2_ detoxification; under high oxidative load, however, peroxiredoxin 2 becomes hyperoxidized and enzymatically inactivated, so that the detoxifying capacity of the cell is progressively exhausted [24,25]. Because these systems are frequently upregulated in tumor cells, their activity is a key determinant of the therapeutic window of CAP [26].

### 2.3. Mitochondrial Dysfunction and the Collapse of Redox Homeostasis

When the RONS load exceeds the capacity of these defenses, the intracellular redox balance collapses, and the mitochondria are a central hub of the resulting injury. Rising intracellular ROS lower the cytosolic pH and perturb calcium handling in intracellular compartments, which in turn disturbs mitochondrial energy metabolism [27]. CAP-induced depletion of glutathione removes a critical protection against oxidative stress and leads to loss of the mitochondrial membrane potential [28]. Impairment of the electron transport chain further increases mitochondrial ROS production, establishing a feed-forward amplification of oxidative damage. The ensuing permeabilization of the mitochondrial outer membrane releases cytochrome c into the cytosol, activating the caspase cascade that executes apoptosis [29,30]. The central role of oxidative stress in this cascade is underscored by the observation that co-treatment with the antioxidant N-acetyl-L-cysteine markedly reduces the number of apoptotic cells [28].

### 2.4. Lipid Peroxidation and Ferroptosis

Beyond apoptosis, CAP-generated RONS drive the peroxidation of polyunsaturated fatty acids within cellular membranes, generating reactive aldehydes such as malondialdehyde and 4-hydroxy-2-nonenal that alter membrane permeability and propagate oxidative injury [19,31]. When lipid peroxidation outpaces the cell’s capacity to repair it, the iron-dependent regulated cell death pathway of ferroptosis is engaged [32]. The principal cellular safeguard is glutathione peroxidase 4 (GPX4), which reduces lipid hydroperoxides and translates the level of oxidative stress into a decision between survival and ferroptotic death [33]. GPX4 activity depends on glutathione, whose synthesis is fed by cystine import through the system xc^−^ transporter (SLC7A11, xCT); accordingly, overexpression of xCT confers intrinsic resistance to CAP, linking ferroptotic vulnerability directly to the xCT–GSH–GPX4 axis [34]. CAP thus appears able to engage several regulated cell death modalities in parallel—apoptosis, necrosis, and ferroptosis—with the predominant route determined by the plasma dose and by the redox and metabolic state of the target cell.

### 2.5. DNA Damage and Activation of Stress Signaling

RONS that reach the nucleus interact with the DNA directly or via intracellular signaling cascades, inducing single- and double-strand breaks and oxidizing nucleobases [19]. These lesions activate checkpoint kinases (CHK1/2) and mitogen-activated protein kinase signaling, together with nuclear translocation of p53, driving cell cycle arrest and, where damage is irreparable, apoptosis [35]. CAP has additionally been reported to elicit epigenetic changes, including altered DNA methylation and histone modification [36].

### 2.6. Extracellular and Membrane-Level Effects

At the cell surface, CAP-derived RONS modify extracellular protein domains and membrane lipids. Lipid peroxidation and oxidation of membrane transport proteins alter membrane permeability, ion channel selectivity, and the osmotic balance between the intra- and extracellular space [31]. These modifications can restrict cell migration, loosen cell–cell and cell–matrix adhesion, and, at high plasma doses, culminate in necrosis [37]. Of particular relevance to the antitumor and immunomodulatory actions discussed below, surface-level oxidation can inactivate catalase and superoxide dismutase—enzymes overexpressed by many tumor cells to shield them from oxidative stress—and can alter antigen presentation in a manner that may enhance immune recognition of treated cells [38,39].

## 3. Types of Plasma Devices

Physical plasma is produced by different types of plasma devices such as plasma jet (APPJ), dielectric barrier discharge (DBD), floating electrode dielectric barrier discharge (FE-DBD), atmospheric pressure glow discharge torch (APGD-t), plasma brush, micro-hollow cathode discharge air plasma jet, microwave plasma torch and nanosecond plasma gun [40]. Another form of plasma is plasma activated medium (PAM) [41]. It refers to the liquid or solution that has been exposed to CAP or its reactive components, resulting in the generation of various reactive species and modification of the chemical composition of the medium. CAP in medicine is generally generated by two main types of devices: (1) direct plasma sources known as DBDs, which use the human body as an electrode; (2) indirect plasma sources such as plasma needles or plasma jets, which generate a discharge between two electrodes [40].

### 3.1. Plasma Jet (or Atmospheric Pressure Plasma Jet—APPJ)

It is an indirect plasma source that uses two coaxial electrodes to create it. A gas is introduced into the space between the electrodes, and the electric field generated by the voltage ionizes the gas, creating a plasma jet. It can be a pure gas, such as oxygen, helium, argon, nitrogen oxides or a mixture of helium and oxygen. The length of the plasma jet varies depending on the gas flow and the applied voltage [40,42]. The temperature of the generated plasma remains stable at room temperature and is about 30 °C at the jet tip. This minimizes the risk of thermal damage to sensitive materials or biological tissues [16,43]. The plasma is generated remotely and the plasma products reach the biological target via a carrier gas [40].

### 3.2. Dielectric Barrier Discharge (DBD)

DBD generates plasma at a distance, similar to plasma jetting. This method consists of ionizing a gas between two electrodes using an alternating current voltage and field strength. The plasma properties depend on several factors, including the applied voltage, the distance between the two electrodes, and the electrode material [42,44]. DBD can also be produced directly on the surface to be treated [42]. In floating electrode dielectric barrier discharge (FE-DBD), the second electrode is made of an active medium, such as skin. This implies that the living tissue or the cell layer itself is used as one of the electrodes directly involved in the plasma process [41]. To effectively treat the surface, the charged electrode must be in direct contact with it and placed within 3 mm [45]. According to Daeschlein et al., there was no significant difference in biological effects between DBD and APPJ in dermatological applications [12,46]. The APPJ device and the DBD are two main CAP devices used in medicine [47].

### 3.3. Plasma-Activated Medium (PAM)

Plasma can trigger a cascade of chemical reactions at the liquid/gas interface, resulting in enrichment of the liquid with RONS, this is known as PAM. The nature and density of these secondary species that interact with cells and tissues are dependent on plasma active species diffusing into fluids [48]. These findings have led to the emergence of a field of research within plasma medicine called “plasma pharmacy,” which focuses on plasma activation of liquids for use in contact with cells and tissues [41].

PAM or plasma-activated water (PAW) is an alternative for situations where plasma generation is not possible, such as in small organs or hard-to-reach cavities. It has shown promising results in a wide range of medical applications, including sterilization, blood coagulation, cancer cell destruction, wound healing, and as a disinfectant for medical equipment and even as a mouthwash for dental problems [49]. PAM remains stable at room temperature and does not damage healthy tissues at effective doses, but it generates reactive species within the tissues of the organism. The efficacy of PAM depends on several factors, such as plasma exposure dose, storage duration, and storage temperature [50,51].

## 4. Problem Statement

CAP has emerged as an emerging technology with therapeutic potential in various medical areas, and its application in ORL could provide new opportunities and solutions to treat a wide range of ORL disorders. For this, it is necessary to fully understand the clinical implications and benefits of CAP in this context, as well as identify specific areas of research needed to fully exploit its potential in ORL.

## 5. Available Bibliographic Information on CAP: Search Strategy and Scope

This article is a narrative review. It aims to provide a broad, critically appraised overview of the possible applications of CAP in ORL rather than a formal systematic synthesis of a narrowly defined question. Given the breadth of the topic—spanning several anatomical regions of the head and neck and multiple therapeutic domains (wound healing, pathogen eradication, immunomodulation, and antitumor treatment) across in vitro, animal, and clinical evidence—a narrative approach was considered the most appropriate framework, and no systematic review protocol was registered.

A comprehensive literature search was conducted in PubMed for records published up to 11 June 2026, returning approximately 3380 records; when narrowed to ORL-relevant sites and indications, 28 reports were identified as directly relevant to ORL and form the core literature analyzed in this review. Search terms were combined with the Boolean operator OR to capture the varied terminology in the field (“cold atmospheric plasma”, “cold atmospheric pressure plasma”, “atmospheric cold plasma”, “cold plasma”, “low-temperature plasma”, “tissue-tolerable plasma”, “non-invasive physical plasma”) and combined with AND with site- and topic-specific terms (“wound healing”, “head and neck cancer”, “squamous cell carcinoma”, “oral cancer”, “nose”, “nasal”, “upper respiratory tract”, “upper airway”).

Titles and abstracts were screened for relevance and full texts assessed where appropriate. Publications are cited either as the core literature analyzed with respect to ORL applications or as supporting references providing background and mechanistic context.

Most of the published articles focused on bacteria or bacterial-infection-related studies (972 publications), followed by general investigations on tumors or cancer (662 articles) and wound healing (295 articles). However, when more specific search terms were used, the number of results fell sharply—to 29 publications for “squamous cell carcinoma” or “oral cancer” and 17 for “head and neck cancer”. In the field of rhinology, combining the search terms with “nose” or “nasal” returned only 13 publications. These findings indicate that there is still a wide field to explore regarding the use of CAP in ORL.

## 6. Cold Atmospheric Plasma Applications

More than a decade ago, the use of plasma sources for medical treatments was approved for the first time in Germany [7]. This was based on the efficacy of CAP to stimulate the tissue regeneration and treatment against microbial pathogens. In addition, CAP has been approved for medical use in the United States and Japan. Numerous studies have demonstrated the efficacy of CAP both in vitro and in vivo [52]. However, it is important to note that only a limited number of these studies have a direct relationship to ORL. Nevertheless, analysis of the results of these studies may provide a basis for potential applications of CAP in the ORL field. Given the breadth and complexity of ORL, it is worthwhile to explore new therapies like CAP.

## 7. CAP for Improving Wound Healing

Cutaneous wound healing is a complex and highly regulated process involving a series of sequential but overlapping events aimed at restoring tissue and homeostatic matrix integrity [53,54]. RONS are essential players in the wound healing process as they control cellular functionality and wound phase transitions [55]. The process starts with the hemostasis/coagulation phase in parallel with an inflammatory phase involving the recruitment of neutrophils, monocytes and macrophages to the site of injury. This is followed by a proliferative phase involving fibroblasts, keratinocytes and endothelial cells, and finally a remodeling phase during which the wound contracts by reorganization of the extracellular matrix [53,54, 56].

A wound with loss of integrity of one or more underlying structures and absence of healing within 8 weeks is defined as a chronic wound [7]. Chronic wounds represent a major challenge in the management of wound healing, as they often do not progress through the phases of wound healing in an orderly fashion [54]. The re-epithelialization process is often impaired in chronic wounds, leading to persistent non-healing wounds [57]. The pathophysiology of chronic wounds is characterized by excessive levels of proinflammatory cytokines and proteases, leading to a proteolytic and degradative wound environment, reduced mitogenic activity and persistent infections [56,57,58].

Improving wound healing in the skin of the face and neck is of paramount importance due to the unique anatomic and esthetic considerations of this region. The delicate and highly visible nature of facial and neck skin requires meticulous healing to minimize scarring, promote optimal cosmetic results, and preserve facial esthetics [59,60]. Rapid and effective wound healing is essential to restore skin barrier integrity, prevent complications such as infection or wound dehiscence, and minimize the risk of functional impairment [59]. Complications related to impaired wound healing, such as infections, delayed wound closure, hypertrophic scars, keloids, or contractures, can significantly affect patient outcomes [60]. These complications can prolong recovery periods, increase pain and discomfort, compromise esthetic outcomes, and cause functional limitations [60,61]. Therefore, optimizing wound healing strategies and implementing appropriate measures to prevent and manage these complications are crucial in ORL.

The application of CAP to an open wound has demonstrated regenerative properties such as increased cutaneous microcirculation [62], stimulation of monocytes [63] and increased keratinocyte proliferation [64]. Keratinocytes play a key role in the restoration of the epidermis through re-epithelialization. To achieve this goal, they undergo significant phenotypic changes, in particular an increase in their proliferation and migration, as well as modification of their differentiation program [56,65]. Helium CAP (<60 s) was shown to accelerate healing in vitro by enhancing keratinocyte migration [56].

CAP has been approved for the treatment of chronic wounds and pathogen-induced skin diseases [66,67]. CAP stimulates the proliferation of various cell types in vitro [68,69,70,71], and may offer a promising therapeutic strategy for chronic wounds controlled by redox reactions. Despite this, the risks involved, the cell types affected and the biological mechanisms involved are not well understood. Contradictory results have been obtained depending on treatment parameters and plasma device settings. For example, some studies showed that CAP treatment increases proliferation and motility of both fibroblasts and keratinocytes [68,72], whereas other studies observed no effect on keratinocytes and only enhanced fibroblast migration [73]. It was suggested that the effect of CAP is dose-dependent, i.e., low doses enhance fibroblast viability and collagen synthesis, whereas high doses inhibit them [74]. Furthermore, opposite effects of CAP on normal versus keloid fibroblasts have been observed [56].

Studies have shown that CAP promotes re-epithelialization and accelerated wound closure in vivo in animal models [73,75,76,77] and in human clinical trials [8,57,78,79]. Based on this, PlasmaDerm^®^ and kINPen^®^ MED were certified in Germany in 2013 as the world’s first plasma devices for the treatment of chronic wounds and pathogen-related skin diseases. They were certified as class IIa medical devices and thus approved for use in hospitals [7]. More recently, a randomized controlled trial by Bakker et al. demonstrated significantly improved wound healing with once- or twice-weekly direct CAP application in chronic venous leg ulcers [9]. A current review (Raissi-Dehkordi et al., 2025) further summarized the molecular pathways by which CAP-generated RONS activate NRF2 signaling, leading to elevated TGF-beta1 levels and reduced MMP-9 expression, thereby supporting granulation tissue formation and suppressing chronic inflammation [80]. However, numerous questions remain about the use of CAP in wound therapy [81].

These collective attributes make CAP a promising adjunctive therapy in ORL, offering the potential to improve wound healing outcomes and enhance patient recovery and satisfaction. Many healing processes can be slowed or hindered by infections resistant to conventional treatments. CAP is not only a therapy that accelerates healing, but also contributes to the eradication of pathogens. CAP generates an antimicrobial environment that reduces wound pathogen load and prevents secondary infections, thus addressing one of the major challenges of healing. By eliminating pathogens and promoting a cleaner wound environment, CAP creates optimal conditions for tissue repair and cell regeneration. This comprehensive approach, combining healing enhancement and pathogen eradication, has the potential to significantly improve outcomes in the wound healing process.

## 8. CAP for the Eradication of Pathogens

Infections by multidrug-resistant bacteria are becoming widespread, necessitating the exploration of non-antibiotic-based therapeutic approaches. Many studies have been conducted on the efficacy of CAP in killing various pathogens [82,83,84]. For example, plasma treatment caused *Bacillus stratosphericus* to lose viability with increasing treatment duration, and *Pseudomonas aeruginosa* to lose viability after five minutes, with no visible biofilm cells after ten minutes of treatment [82,85]. In both planktonic and biofilm forms, *Escherichia coli*, *Listeria monocytogenes* and *Staphylococcus aureus* showed significant population reduction and widespread inactivation after CAP treatment [82,83].

Within the oral cavity context, a systematic review has documented CAP applications in prosthodontics, encompassing prosthetic surface decontamination, implant-associated biofilm eradication, and mucosal wound healing—all of direct relevance to ORL practice given the frequent overlap of head and neck oncological and reconstructive procedures with oral rehabilitation [13].

CAP has been shown to improve wound healing in infections with methicillin-resistant *Staphylococcus aureus* strains [86,87]. A study by Lunder et al. confirmed that CAP achieves up to 5.24 log10 cfu/cm^2^ reduction in MRSA biofilms of varying maturity within 180 s, without inducing treatment resistance [88].

Further reduction in fungal and bacterial colonization is considered promising [12]. At present, the limiting factor appears to be wound size [78]. CAP is a promising approach that may prove effective against these challenging pathogens. CAP has been shown to exhibit antimicrobial activity against a broad spectrum of microorganisms [12,89,90]. A 2025 review by Ding et al. comprehensively summarized CAP disinfection mechanisms and its efficacy against clinically important bacteria including *P. aeruginosa*, *K. pneumoniae*, and MRSA, highlighting its potential role in addressing antimicrobial resistance [91].

Plasma-activated liquids have further demonstrated efficacy against *Helicobacter pylori* and resistant hospital pathogens including ESKAPE organisms through a combination of acidification and reactive oxygen and nitrogen species, broadening the antimicrobial spectrum of CAP-based strategies beyond wound-associated bacteria [92] (ESKAPE: Acronym for *Enterococcus faecium*, *Staphylococcus aureus*, *Klebsiella pneumoniae*, *Acinetobacter baumannii*, *Pseudomonas aeruginosa*, and *Enterobacter* species).

Due to its antimicrobial and immunomodulatory effects, CAP is a potential tool for the treatment of infections and inflammations in rhinosinus and other areas of ORL. Notably, CAP treatment of actinic keratosis lesions selectively reduced the abundance of *Staphylococcus* species—a genus overrepresented in premalignant and malignant skin lesions—as compared with diclofenac 3% gel, an established topical field-directed therapy, suggesting an additional antimicrobial dimension to CAP’s therapeutic action in premalignant skin disease [93].

## 9. Microbial Pathogens and Associated Complications in Nasal Cavity Infections

Like many anatomic structures, the nasal cavity harbors a wide range of microbiota and serves as a portal of entry for different pathogens. Although a healthy adult may carry certain bacterial species asymptomatically within the nasal cavity, disruption of this environment can lead to significant infections and complications [94].

*Staphylococcus aureus* (*S. aureus*), *Streptococcus pneumoniae* (*S. pneumoniae*) and *Haemophilus influenzae* (*H. influenzae*) are bacteria known to colonize the nasal cavity of adults. Although this asymptomatic colonization can, under certain circumstances, become harmful and lead to local soft tissue infections (*S. aureus*), pneumonia (*S. pneumoniae*) or chronic rhinosinusitis (*H. influenzae*), it can also progress to life-threatening bacteremia or translocate to other sterile areas, which can lead to meningitis [94,95]. Furthermore, bacterial colonization causing rhinitis and rhinosinusitis has been observed to induce biofilm formation on the nasal mucosa, which disrupts the mucosal barrier and leads to chronic inflammation, recurrent infections and antibiotic resistance [96]. This phenomenon manifests especially with colonization of the nasal cavity by *S. aureus* and has been associated with the development of a more severe phenotype of chronic rhinosinusitis with nasal polyps (CRSwNP) [97]. Other pathogens are also detected in nasal washings of patients with chronic rhinosinusitis, namely viruses of the rhinovirus and influenza families, which may be a cause of exacerbation of underlying respiratory conditions [98,99].

Consequently, persistent disruption of the nasal microbiome and exposure to pathogens contribute to the development of chronic rhinosinusitis, characterized by persistent inflammatory conditions of the sinus mucosa. The treatment of nasal sinus infections by novel antimicrobial therapeutic approaches is a critical step in preventing the spread of asymptomatic or localized infections and combating biofilm production.

## 10. Improved Wound Healing with Simultaneous Germ Eradication Through the Use of CAP

This potential to enhance wound healing and, at the same time, eradicate germs can be exploited in the operation area. Direct exposure of the wound or operation area to CAP can help reduce the risk of infection, which is a crucial factor for successful wound healing. A great example of this is the effect of CAP on *Staphylococcus aureus*.

*S. aureus* is one of the common bacteria capable of causing a variety of infections in the head and neck region, including infections of the skin, mucosa and soft tissues [100]. *S. aureus* serine protein-like proteases (spls) are among the virulence factors of the bacterium that can modulate host type 2 immunity [101]. A strong connection between *S. aureus* virulence factors and modulation of the immune response, in particular the effect of *S. aureus* proteases on the regulation of the inflammatory cytokine IL-33, has so far been established in the literature [102]. IL-33 plays an important role in immune responses, including inflammation and tissue repair, depending on its localization within the cell nucleus or cytoplasm [103]. Cytoplasm-available IL-33 is cleaved into a mature active proinflammatory form by *S. aureus* proteases [104]. Thus, cleaved IL-33 increases local and systemic inflammation and thus significantly influences wound healing. It has been shown that CAP treatment causes a cytoplasmic decrease and increase in internuclear IL-33 [105]. The antibacterial role of plasma is expressed through stimulation of the immune system.

Multiple other elements interacting in the process of wound healing and germ eradication through CAP can also be described. The stimulation of RONS production by CAP is involved in cell signaling and may contribute to wound closure and tissue regeneration (Figure 1) [6,106]. CAP also modulates the expression of growth factors, such as transforming growth factor beta (TGF-β), vascular endothelial growth factor (VEGF) and fibroblast growth factor (FGF), which are essential for tissue repair and angiogenesis [107].

In addition, CAP can induce a controlled inflammatory response, promoting the recruitment of immune cells to the wound site. This may enhance debris clearance and facilitate the healing process. CAP has also been shown to stimulate nitric oxide (NO) production, which is involved in wound healing by promoting vasodilation and improving blood flow [106].

Expanding the evidence for wound-relevant applications, plasma-activated water (PAW) has recently demonstrated potent antibiofilm activity against MRSA-infected burn wounds in a murine model, achieving a significant reduction in bacterial load and markedly improved wound re-epithelization compared to vehicle-treated controls [108]. These results support the translational potential of PAW as a topical microbial wound cleanser and a phase I clinical trial is currently planned.

Overall, the use of CAP in ORL wound healing offers a multifaceted approach, combining antimicrobial effects, the promotion of wound healing processes, and improved disinfection.

## 11. Modulation of the Immune Response by CAP

Immunological interaction plays a crucial role in wound healing. Following the application of CAP to wounds, an immune response occurs involving the activation of immune cells, release of inflammatory mediators and modulation of growth factors. These events trigger a series of cellular responses, such as inflammatory cell migration to the wound site, fibroblast proliferation and new tissue formation. In addition, CAP can modulate the immune response by regulating the expression of key molecules, such as cytokines, chemokines, and transcription factors, which are involved in immune cell–cell communication and coordination of wound healing [106].

Understanding these immunological effects of CAP is crucial for understanding its antitumor effect. These immunological effects are of great importance in the context of its anticancer action and support its potential as a multi-targeted therapeutic tool in ORL.

CAP has been shown to modulate the immune response in several ways:

### 11.1. Activation of Immune Cells

CAP treatment has been shown to activate several immune cell populations involved in antitumor immune responses. Macrophages, dendritic cells and natural killer cells play a key role in immune surveillance and tumor killing [107,109]. CAP induces macrophage polarization toward an antitumor M1 phenotype, leading to the secretion of cytokines (IL-1α, IL-1β, IL-6, TNF-α, etc.) that can directly kill tumor cells [107,110,111]. In addition, CAP promotes the maturation of dendritic cells, enhancing their antigen-presenting capacity and enabling robust T-cell activation [112]. Moreover, CAP stimulates the activity of natural killer cells, increasing their ability to recognize and eliminate tumor cells [113,114].

### 11.2. Induction of Immunogenic Cell Death (ICD)

ICD is a form of programmed cell death characterized by the release of damage-associated molecular patterns (DAMPs), which act as danger signals to activate the immune system [107,115]. CAP treatment triggers ICD in tumor cells, resulting in the release of DAMPs such as ATP, calreticulin and heat shock proteins, HSP70 and HSP90 [116,117]. These DAMPs attract immune cells to the site of cell death, facilitating recognition and phagocytosis of tumor cells by antigen-presenting cells. These CAP-induced DAMPs enhance the immune response against tumor cells, potentially contributing to tumor regression and long-term immune memory [107,118]. The combination of CAP with pulsed electric fields has been shown to synergistically enhance ICD marker expression, suggesting that multimodal physical strategies may amplify antitumor immune priming [119].

### 11.3. Release of Cytokines and Chemokines

CAP treatment stimulates the release of cytokines and chemokines, which regulate the immune response and promote antitumor activity [120]. These soluble factors attract immune cells to the tumor microenvironment, enhancing their activation and recruitment. The resulting infiltration of immune cells may result in an amplified antitumor immune response [110,111,118]. In addition, CAP-induced cytokines may exert direct cytotoxic effects on the tumor cells, further contributing to tumor cell death [107,111,120].

### 11.4. Modulation of Immune Checkpoints

Programmed cell death protein 1 (PD-1) is a key immune inhibitory receptor that is predominantly expressed on T-cells and plays an important role in a variety of physiological phenomena, including infection, cancer and immune homeostasis [112]. PD-L1, the cognate ligand of PD-1, shows elevated expression levels in numerous types of cancer, most notably head and neck cancers [121]. Consequently, the use of antibodies directed against the PD-1/PD-L1 interaction has emerged as a promising strategy to prevent tumor progression [122,123]. CAP treatment has been shown to modulate PD-L1 expression in tumor or immune cells, which may increase the efficacy of immune checkpoint blockade therapies. This modulation may release the suppressed immune response [121,124]. Validating these findings, Wang et al. demonstrated that CAP upregulated PD-L1 expression in HNSCC cells and significantly increased CD8+ and CD4+ T-cell tumor infiltration, with synergistic tumor suppression when combined with anti-PD-1 immune checkpoint blockade therapy [125].

### 11.5. Stimulation of the Immune System Through Interleukins

One of the immunomodulatory effects of CAP is the cytoplasmic decrease and intranuclear increase in IL-1 beta and IL-33 [105]. Studies have shown that IL-33 is expressed in a variety of cells, including endothelial cells, fibroblast cross-linked cells, and epithelial cells, while acting as a dual function protein. When found in the cytoplasm, IL-33 can function as a proinflammatory cytokine, while in the nucleus it has transcriptional repressor properties. Thus, the role of IL-33 differs depending on its location within the cell [103].

In previous work on the effect of CAP on respiratory epithelial wounds, immune stimulation and modulation was established for different cytokines after CAP treatment. Total cytosolic concentrations of IL-1beta and IL-33 decreased in cell lines representative of healthy respiratory epithelium after CAP treatment. Immunoblotting of intracellular cytosolic concentrations of full-length IL-1beta and IL-33 revealed a decrease in these pro-interleukins in bronchial epithelial S9 cells 30 min and 48 h after CAP treatment [105].

CAP treated wounds achieved better wound healing results compared to untreated control wounds [105,126].

The effects of CAP on wound healing and pathogen eradication, together with its inherent ability to modulate the immune system, are also highly beneficial in the context of tumor pathology. CAP has proven to be a promising therapy in the treatment of cancer [52]. In the field of head and neck tumor, the potential of CAP as an adjuvant therapy to enhance the immune response against tumor cells and stimulate apoptosis and cell death has been investigated [127]. These properties of CAP offer new opportunities, where its application may contribute to improve therapeutic outcomes and offer effective alternatives for the treatment of head and neck cancer.

## 12. CAP as Adjuvant Therapy in Head and Neck Cancer

### 12.1. Effects of CAP on Tumor Cells

The antitumor effect of CAP has been demonstrated in various types of cancer cell lines in vitro, such as melanoma, glioblastoma, lung cancer, colon cancer, pancreatic cancer, breast cancer, leukemia, thyroid cancer, and head and neck cancer [52,128,129,130]. Neoplasms of different entities show reduced cell proliferation, adhesion and migration and increased apoptosis after treatment with CAP [131]. A comprehensive review in 2025 has systematically summarized the mechanisms of CAP-mediated anticancer effects—including apoptosis, immunogenic cell death, pyroptosis, ferroptosis, and tumor immune microenvironment modulation—and outlined advances in delivery systems ranging from direct application to hydrogel-based and microneedle-assisted approaches [32].

In cancer, malignant transformation often disrupts normal cellular function, leading to an intensification of cellular redox processes [132]. Redox imbalance refers to an abnormal state in which the effects of oxidants and antioxidants are altered [133,134]. It has been shown from experiments in melanoma, prostate carcinoma, glioblastoma and breast carcinoma that plasma effects depend on the duration of treatment [37,135,136]. In some cases, there was only a significant reduction in tumor cell burden in vitro after a treatment duration of 2.83 s/cm^2^ (150 s/53 cm^2^) [136]. Walk et al. were able to almost double the survival time with CAP treatment of the neuroblastoma in mice [137]. Cell surface receptors play a key role in sensitizing cancer cells to various external stimuli. However, many therapies fail due to mutations in the target receptors. This has led to the development of dual-target strategies, such as the combination of venetoclax and rituximab for the treatment of refractory chronic lymphocytic leukemia [138,139]. The extension of such strategies by means of a CAP treatment to address multiple target structures is promising.

Early approaches to in vitro treatment with CAP showed a dependence of the response on the mutational status of degenerating cells, in addition to a reduction in tumor mass [140,141,142,143]. It may affect mitochondrial function, increase oxidative stress and alter cellular energy production. These metabolic changes may contribute to inhibition of tumor cell growth and survival [47]. CAP can induce apoptosis specifically in cancer cells, resulting in the elimination of cancer cells while minimizing damage to healthy tissues [121,144]. The differential roles of short-lived and long-lived ROS in mediating CAP selectivity toward malignant cells have been characterized by Sklias et al., underscoring that the RONS profile of the plasma source critically determines its therapeutic window [145]. The interplay of Nrf2/NF-κB signaling in malignant versus non-malignant dermal cells after CAP stimulation provides a molecular framework for this selectivity [146].

CAP treatment can activate apoptotic signaling pathways in cancer cells. A key pathway involved is the intrinsic or mitochondrial pathway of apoptosis. DNA damage and mitochondrial dysfunction induced by CAP trigger activation of proapoptotic proteins, such as Bax and Bak, and inhibition of antiapoptotic proteins such as Bcl-2 [40,147]. This disruption of the balance between pro-apoptotic and anti-apoptotic proteins promotes cytochrome c release and subsequent activation of caspases, ultimately leading to apoptosis [148]. CAP has been described to exhibit anti-angiogenic properties in cancer cells. It can inhibit the formation of new blood vessels, thus limiting the blood supply to the tumor and preventing its growth and metastasis [149].

CAP has demonstrated its potential in the treatment of several types of head and neck cancers, including squamous cell carcinoma (SCC) of the upper aerodigestive tract (UAD), and skin cancers, such as basal cell carcinoma (BCC) and malignant melanoma, as well as for glandular cancers of the head and neck. CAP treatment has been shown to selectively eliminate cancer cells without affecting healthy cells, making it a promising therapeutic option [121]. A systematic review by Perrotti et al. encompassing 24 preclinical and clinical CAP studies in HNC highlighted the urgent need for device and protocol standardization as a prerequisite for clinical translation, while confirming the consistent antitumor activity of CAP across HNC entities [150].

### 12.2. CAP as a Treatment for Head and Neck Skin Cancer

Skin cancers are frequently located in sun-exposed regions of the head and neck, which can result in significant morbidity during their diagnosis and treatment [151]. Among head and neck skin cancers, BCC is the most prevalent, followed by SCC and malignant melanoma. To explore the potential of CAP for treating cutaneous SCC of the head and neck, insights may be drawn from successful CAP applications in upper aerodigestive tract SCC, given their shared squamous histology [121]. The mechanistic rationale for plasma jet application in skin cancer treatment, including differential tissue penetration of reactive species and the physical determinants of selectivity, has been systematically reviewed by Bekeschus et al. [152].

Melanoma accounts for only 3% of skin cancer, but is responsible for 65% of skin cancer-related deaths [153]. It has been shown that different tumor cell types have different sensitivity to RONS and, therefore, different sensitivity to CAP treatment [34]. For this reason, screening prior to CAP treatment in melanoma is recommended. CAP in melanoma can be performed by direct or indirect exposure and in combination with other therapies [121]. In direct application of CAP to melanoma cells it can induce DNA damage, leading to cell cycle arrest and apoptosis [154]. CAP also inhibits cell migration and adhesion in melanoma cells and reduces tumor cell motility and colony formation [155]. Indirect therapy with CAP, such as PAM, has comparable antitumor efficacy and can access deep tumors and regional metastases by injection [156]. Combining CAP with other oncologic therapies, such as chemotherapy or electrochemotherapy, has been shown to improve tumor control and increase survival rates [156,157]. Synergistic effects have also been observed when CAP is combined with nanomedicine, resulting in mitochondrial damage [158].

An ORL-focused narrative review has further synthesized current evidence on CAP applications across skin cancer entities of the head and neck, emphasizing the translational potential of plasma dermatology within the ORL context [159].

In vivo confirmation of CAP efficacy against cutaneous SCC and melanoma was provided by multimodal imaging (MRI and [18F]FDG PET/CT), demonstrating increased intratumoral reactive species and reduced tumor growth upon CAP treatment in xenograft models [160].

In the case of BCC, although limited studies have explored treatment with CAP, PAM generated by CAP irradiation has shown potential to decrease cell viability and induce apoptosis in BCC cells. Changes in MAPK and TNF signaling pathways have been implicated in PAM-induced apoptosis in BCC cells [121,161]. Selective inhibition of cutaneous SCC (A431) by CAP-activated medium has been demonstrated in vitro, with preferential cytotoxicity towards tumor cells compared to normal keratinocytes and a dose-dependent apoptotic response [162].

### 12.3. CAP as a Treatment for Squamous Cell Carcinoma of the UAD

In SCC, the selectivity of CAP for cancer cells can be attributed to factors such as cell cycle manipulation, cell membrane differences and cell receptor interactions. Optimized adaptation to RONS occurs through activation of redox systems due to increased basal metabolic rate and proliferation [134]. Tumor cells, which usually have a higher proliferation rate and a higher proportion of cells in the S-phase of the cell cycle, are more susceptible to CAP treatment [161]. In addition, differences in cell membrane structure, including higher expression of aquaporin proteins in tumor cells and lower cholesterol content, contribute to the selective vulnerability of cancer cells to CAP-induced oxidative stress. Excessive oxidative stress can alter cellular redox balance and trigger apoptosis [121,163]. It can lead to the activation of stress-responsive kinases and the generation of lipid peroxidation products that promote apoptotic signaling [121,148]. In addition, overexpression of epidermal growth factor receptor (EGFR) in SCC cells allows CAP to induce EGFR dysfunction, leading to selective cancer cell death [156].

Extending these findings, recent studies have demonstrated that CAP exerts potent anti-OSCC effects via diverse molecular pathways, including p53 pathway activation [164], focal adhesion kinase (FAK) inhibition in combination with gold nanoparticles [165], synergistic cytotoxicity with nanohydroxyapatite [166], and enhanced chemosensitivity in both 2D and 3D tumor models [127].

Beyond direct cytotoxicity, argon-based CAP jet irradiation has been shown to downregulate oncogenic miRNAs including miR-21 and miR-31 in oral carcinoma cells [167], while plasma-activated medium derived from no-ozone CAP selectively triggers apoptosis through MAPK pathway activation [168].

Translationally, the antitumor activity of CAP has also been demonstrated in feline oral SCC models, which share key molecular features with human OSCC and provide a relevant comparative oncology platform for evaluating plasma-based therapies [169].

Indirect CAP treatment via plasma-activated medium has also demonstrated selective cytotoxicity in HNSCC models, with efficacy depending on culture medium composition [170] and confirmed across multiple HNSCC cell lines under patient-relevant conditions [171].

Cold physical plasma has further demonstrated an anti-inflammatory effect in oral leukoplakia tissue ex vivo, suggesting potential utility in the management of oral potentially malignant disorders—a clinically significant premalignant condition frequently encountered in ORL practice [172].

### 12.4. CAP as a Treatment for Glandular Head and Neck Cancer

Thyroid cancers, which include several subtypes such as papillary, follicular, medullary, anaplastic, and thyroid lymphoma, can often not be cured by surgery alone [121]. CAP has been shown to be selective with thyroid cancer cells, but not with healthy cells. It induces an increase in RONS levels in thyroid cancer cells, leading to apoptosis [136]. In addition, CAP decreases metabolic viability and colony formation of thyroid cancer cells [173]. Studies have identified possible molecular mechanisms underlying the selectivity of CAP, such as upregulation of early growth response gene 1 (EGR1) expression, which increases the level of DNA damage-inducible protein 45a (GADD45A) and inhibits invasion and metastasis of papillary thyroid cancer cells by modulating the expression of MMP2/MMP9 and UPA proteins [173].

In salivary gland cancers, particularly those arising in the parotid gland, CAP has also been shown to increase cancer cell apoptosis. There are data reporting increased RONS in thyroid cancer cells following CAP application. CAP may contribute to cell apoptosis through alterations in the GSH/GSSG ratio, the NADP^+^/NADPH ratio and total antioxidant activity [136]. However, the specific mechanisms underlying this effect have not been thoroughly investigated. It remains to be determined whether the antineoplastic effect of CAP on parotid tumor cells shares similar mechanisms observed after CAP treatment of head and neck SCCs of the upper aerodigestive tract [121].

## 13. CAP in Rhinology

Few studies exploring the use of CAP have been performed in the field of rhinology. On the one hand, the previously presented data on the effects on rhinosinus infections support the ability of CAP to kill bacteria, especially with nasal colonies of *S. aureus* [83,97]. Another study investigated the efficacy of CAP in preventing the entry of SARS-CoV-2 virus into cells. The results showed that CAP effectively inhibited virus entry in both in vitro and in vivo experiments. Treatment with CAP triggered rapid internalization and nuclear translocation of the ACE2 virus receptor, with hydroxyl radicals (-OH) being the most effective CAP components in triggering this response. The study suggests that CAP could be developed into products such as nasal/mucosal sprays to help control SARS-CoV-2 and other ACE2-utilizing viruses [174]. Complementarily, PLASMASKOP, a miniaturized neon plasma jet developed in a joint cooperative project to enable endoscopic plasma application within the anatomically complex nasal cavity, was subsequently evaluated by Mrochen et al. and shown to exert antiviral activity against a murine coronavirus model, achieving up to a 3000-fold reduction in viral infectivity. The greater potency observed in the conductive treatment mode further supports its potential as a nasal antiviral treatment modality [175]. The technical development of PLASMASKOP is described in more detail in Section 15.

Another area in which CAP has been investigated is nasal mucosal healing. CAP has been shown to have promising potential for promoting nasal mucosal wound healing following surgery or trauma. Its use as adjuvant therapy in surgical wound healing has been explored, as well as in the treatment of chronic ulcers or injuries in these specific areas [176]. Importantly, although CAP therapy shows promise, further research is needed to establish optimal treatment protocols, evaluate long-term efficacy, and ensure safety in clinical settings. Throughout this work, the potential of CAP in multiple aspects is evident, which is beneficial to apply in the area of Otolaryngology.

Beyond direct antimicrobial and wound healing applications, CAP has been explored as a surface modification tool for drug-delivery microspheres targeting the nasal septum, offering a novel approach to localized pharmacotherapy within the nasal cavity [177].

A recent clinical application of helium-based cold plasma (J-Plasma) has been reported for the treatment of rhinophyma, representing one of the first direct clinical uses of CAP technology at the nasal surface and demonstrating safe and effective tissue ablation without thermal damage to surrounding structures [178].

**Figure 2:**
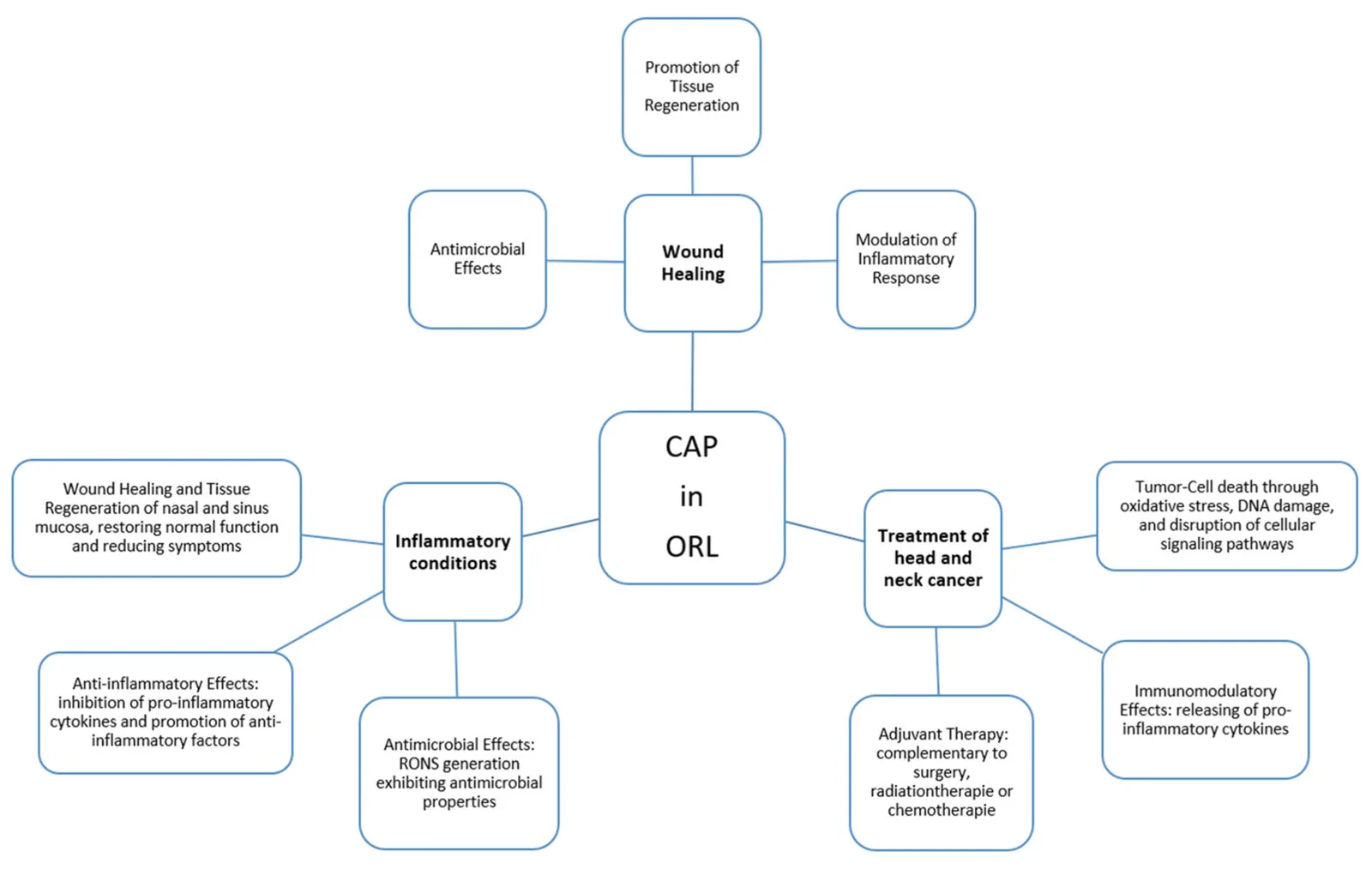
Possible applications of cold atmospheric plasma (CAP) in otorhinolaryngology (ORL).

One of the predominant challenges in the application of plasma technology in the field of Otolaryngology is the intricate anatomy, especially in Rhinology. Existing instruments designed for CAP application often pose limitations due to their considerable size and impracticality for endoscopic procedures. The pressing need for miniaturization becomes apparent, as it is indispensable to unlock the full potential of CAP in both Otolaryngology and Rhinology (Figure 2).

## 14. CAP in Upper Respiratory Tract

Beyond the nasal cavity, a series of recent studies have begun to explore CAP across the wider upper respiratory tract (URT), motivated by the prevention of ventilator-associated pneumonia and respiratory viral infection in intubated patients. Using a surface micro-discharge device in a URT model, Karrer et al. reported that a five-minute CAP treatment at pharyngeal and subglottic positions produced no significant cytotoxic or molecular changes in human oral keratinocytes, bronchial–tracheal epithelial cells, or lung fibroblasts [179]. In a complementary three-dimensional URT model, Reichold et al. characterized the response of polymorphonuclear neutrophils to CAP, aiming for antimicrobial and immunostimulatory benefit without provoking an excessive inflammatory reaction [180]. Extending these findings in vivo, Arndt et al. exposed wildtype mice to single and repeated CAP sessions with the same device and assessed immune cell responses and long-term effects, providing early evidence on the tolerability of repeated URT exposure [181]. Together, these studies extend the potential ORL applications of CAP beyond the nasal cavity toward the pharynx and subglottis, while beginning to address the safety and immunological questions on which clinical adoption will depend.

## 15. Future Technical Implementations

### 15.1. Requirements for Miniaturization of Plasma Sources

The development of innovative CAP delivery techniques or the combination of CAP with other treatment modalities that can enhance its penetration into deeper layers may help overcome the limitation of superficial tissue penetration. This could involve methods such as optimizing plasma jet design, exploring different plasma sources, or using physical or chemical agents to facilitate the reach of CAP into deeper tissues.

### 15.2. Miniaturization of Plasma Sources

The miniaturization of CAP sources would benefit the field of Otolaryngology and very significantly Rhinology. Firstly, it would allow improved accessibility for targeting specific areas. Secondly, it can offer greater precision and localization, allowing treatment to be focused while minimizing risks to nearby vital structures, such as nerves or vascular structure. Thirdly, its adaptability to cavities would facilitate safe and effective treatment in confined spaces. In addition, miniaturization prioritizes patient comfort by reducing intrusion and trauma, resulting in shorter treatment times and greater therapeutic compliance. Finally, it improves practicality and portability, making CAP sources easier to handle and integrate into clinical settings. Therefore, the miniaturization of CAP sources is crucial for safe, accurate and effective treatment, optimizing therapeutic outcomes in the face of challenging ORL anatomical considerations.

Notably, the first FDA-approved Phase I clinical trial of intraoperative CAP was completed in 2021, demonstrating safety and promising tumor response rates in patients with advanced solid tumors, lending important clinical translational momentum to device development efforts [15].

### 15.3. Combination with Endoscopes

The concept of endoscopic CAP delivery was pioneered by Robert et al., who demonstrated first in vivo antitumor effects of a plasma gun system in orthotopic colorectal and pancreatic cancer mouse models, and achieved plasma delivery to the lung via a catheter-based endoscopic protocol—establishing the feasibility of intra-corporeal CAP application [182].

**Figure 3:**
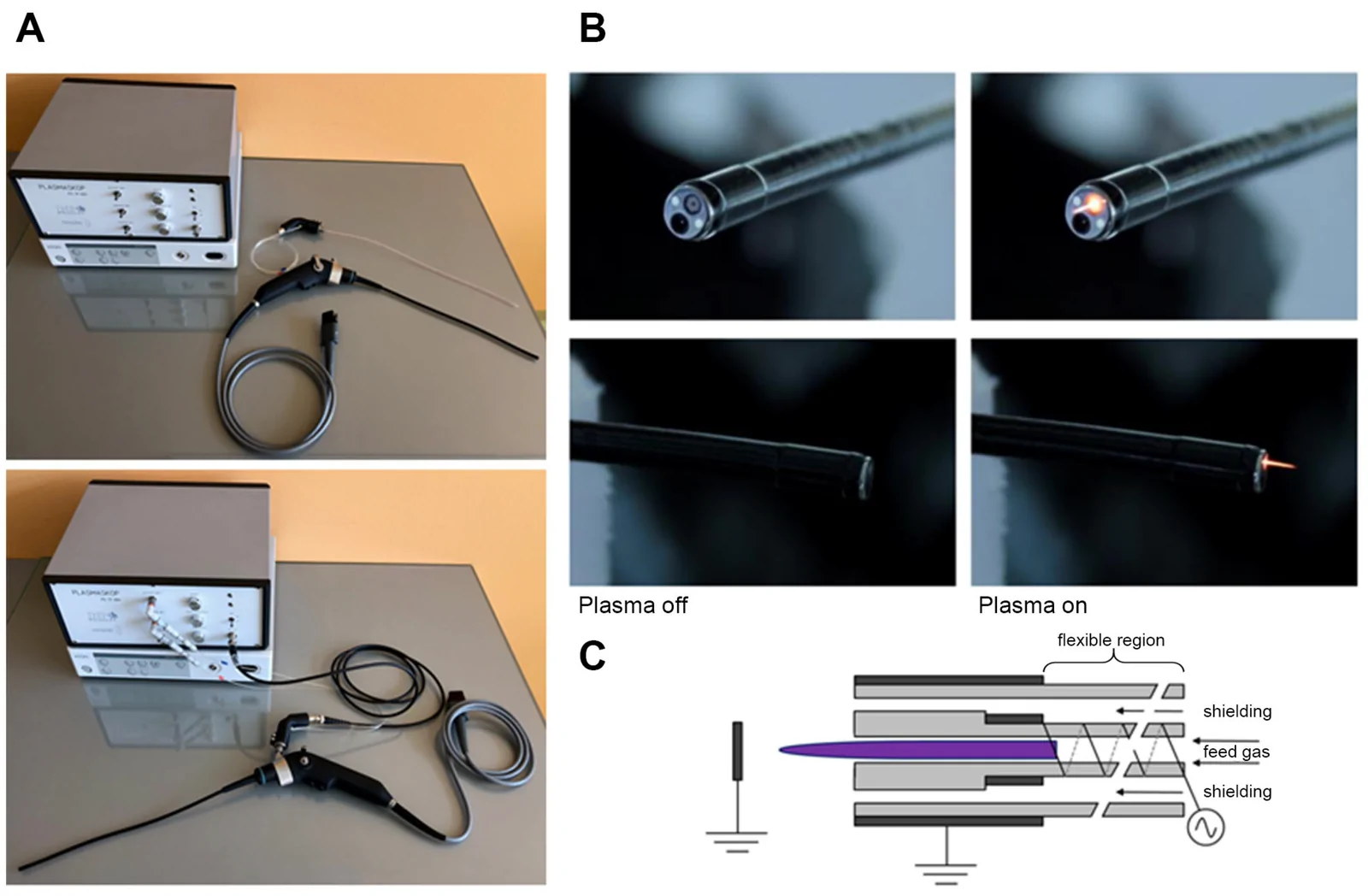
The endoscope-based CAP device for ORL applications (PLASMASKOP): (**A**) disassembled and assembled PLASMASKOP; (**B**) the distal end of the PLASMASKOP device with neon plasma jet; (**C**) a sketch of the plasma source for endoscopic applications. Sources: ORL Research Laboratory of the University Medicine of Greifswald, Germany and reprint from [183] under CC BY-NC-ND 3.0.

Based on the benefits and the need to fully utilize the potential of CAP, we developed in a joint cooperation project of the ORL laboratory at the University Medicine of Greifswald (Greifswald, Germany), plasma source developers (INP Greifswald, Germany) and industrial partners (neoplas GmbH, Greifswald, Germany and Xion GmbH, Berlin, Germany) the first endoscope-based atmospheric pressure plasma jet (PLASMASKOP) funded by the German Federal Ministry of Education and Research (Figure 3).

The foundational engineering work enabling the PLASMASKOP was established by Winter et al., who demonstrated that a coaxial shielding gas channel is essential to maintain stable plasma jet formation in semi-closed body cavities, and that narrow electrode windings effectively suppress unwanted in-tube discharges [184]. This device combination achieves greater than 5-log bacterial reduction against *Pseudomonas aeruginosa* while maintaining patient leakage currents well below the EN 60601-1 safety threshold—demonstrating both biological efficacy and electrical safety for endoscopic use [185]. Among the tests performed to date, the physiological and molecular effects of PLASMASKOP CAP treatment on various human epithelial and tumor cell lines were analyzed by kinetic analysis, FACS, COMET-Assays, Proteome and Western blot analysis (unpublished data). The initial results of physiological and molecular biological studies of the PLASMASKOP—conducted in both neon and helium modes and focusing on the effects on wound healing and adjuvant tumor treatment—are very promising and will be the subject of future publications.

Quality control and reproducibility between individual devices are critical prerequisites for safe clinical implementation; a comparability study of those miniaturized neon plasma jets demonstrated that dissipated electrical power combined with liquid-phase reactive oxygen species measurement provides a reliable quality control framework for endoscopic plasma device series production [186]. Importantly, comprehensive start-up analysis of those miniaturized helium plasma jet has shown that stable and reproducible reactive species output—a prerequisite for standardized dosimetry—is only achieved after a defined warm-up period of approximately 12–15 min, with implications for the design of reliable clinical treatment protocols [187].

Importantly, miniaturization of CAP devices for endoscopic surgeries is an active area of research, and both development and adoption of these devices may be in the early stages. Continued technological advances and further studies are required to optimize miniaturized CAP devices for clinical endoscopical use.

## 16. Critical Appraisal and Current Limitations

Despite the breadth of evidence summarized in the preceding sections, several limitations temper the enthusiasm for CAP in ORL and should be weighed before its potential can be considered established.

The most pervasive difficulty is the heterogeneity of the treatment conditions employed across studies. Reports vary substantially in terms of plasma source (including dielectric barrier discharge, plasma jet, or plasma-activated liquid), feed gas, applied voltage and frequency, treatment distance, and exposure duration. Moreover, only a limited number of studies provide a standardized and physically characterized dose. Consequently, treatments that are nominally comparable may generate markedly different reactive-species profiles, limiting the comparability of outcomes across studies and, in some cases even between different devices within the same study. This lack of harmonized dosimetry is widely regarded as one of the principal barriers to clinical translation [150], and it qualifies almost every cross-study comparison drawn in the preceding sections. Recent work to define reliable quality control and start-up parameters for miniaturized jets [186,187] has therefore been an essential precondition for the reproducibility that clinical adoption will require.

This heterogeneity also explains much of the apparent inconsistency in the wound healing literature. The seemingly conflicting reports—CAP enhancing keratinocyte proliferation in some studies and having no such effect in others—are best understood not as genuine disagreement but as a consequence of the biphasic, dose-dependent nature of CAP. Low doses, with correspondingly low RONS concentrations, tend to stimulate the proliferation and migration of fibroblasts and keratinocytes and to promote angiogenesis, whereas higher doses inhibit these processes and ultimately become cytotoxic [74]; the opposing effects reported on normal versus keloid fibroblasts illustrate the same principle at the level of the target cell. The practical implications are substantial: the therapeutic efficacy of CAP cannot be inferred from the technology itself but depends on a carefully defined treatment window that must be empirically established for each clinical indication and device configuration. Current studies, however, often lack the methodological standardization and detailed reporting required to adequately define such treatment windows.

Similar caution is warranted regarding the antitumor evidence, which requires careful interpretation and contextualization. The great majority of studies demonstrating CAP-induced cytotoxicity, apoptosis, and selectivity have been conducted in vitro, frequently in a limited set of established cell lines, with fewer animal studies and only isolated clinical reports [52]. The most substantial clinical experience in head and neck cancer remains a small palliative series [130], and the first Phase I trial addressed mixed advanced solid tumors rather than an ORL-specific cohort [15]. Findings obtained under highly controlled two-dimensional culture conditions cannot be directly extrapolated to the complex three-dimensional tumor environment, where tissue architecture, perfusion, extracellular matrix interactions, and immune components critically influence therapeutic responses.

Finally, the selectivity of CAP for malignant cells—although consistently reported—is not absolute. Sensitivity varies considerably between tumor entities and even between cell lines of the same entity, and intrinsic resistance mechanisms, such as high xCT (SLC7A11) expression or particular TP53 mutational states, can substantially attenuate the response [34]. The magnitude of the therapeutic window between malignant and healthy tissue is therefore likely to be context-dependent rather than a fixed property of CAP, which reinforces the case for pre-treatment characterization of the target tissue.

A further pattern deserves emphasis, as it shapes how the sparse ORL-specific evidence should be read. As noted in Section 5, the number of available publications declines steeply as the target site becomes anatomically less accessible—from the readily reachable oral cavity, through the head and neck region generally, to the confined nasal and paranasal spaces, where only a handful of studies exist (29 versus 13 records, respectively). Although this gradient is certainly influenced by other factors—including the well-established oral medicine and dental research communities and a degree of circularity, whereby the absence of suitable delivery devices has itself limited study of the least accessible sites—anatomical accessibility appears to be a genuine rate-limiting constraint on the field. Conventional CAP applicators such as the kINPen MED cannot be introduced into the narrow cavities of the nose, paranasal sinuses, or larynx, so the very regions in which ORL might most benefit from CAP are those for which almost no evidence yet exists. This reframes the scarcity of ORL-specific data not merely as a gap in the literature but as a direct consequence of a technological limitation. It also identifies the development of miniaturized, endoscope-integrated plasma sources—of which the PLASMASKOP described in Section 15 is one example—as the key enabling step: by making these previously unreachable sites accessible, such devices are the precondition for generating the clinical evidence the field currently lacks, and the ORL-specific literature is likely to expand as they mature.

## 17. Conclusions

CAP represents a promising and multifaceted therapeutic modality in otorhinolaryngology. With its capacity to modulate redox signaling, eradicate pathogens, enhance wound healing, and trigger immunogenic tumor cell death, CAP has demonstrated substantial preclinical and early clinical efficacy across a range of ORL applications. However, its clinical translation remains at an early stage, and several critical questions must be addressed to facilitate routine implementation.

Future research should clarify the optimal CAP dosimetry for different tissue types and clinical indications, particularly in relation to treatment duration, frequency, and gas composition. A better understanding of how CAP-induced immune modulation varies across mucosal environments, and how it might be harnessed to potentiate local or systemic antitumor responses, is essential. Furthermore, the identification of redox-sensitive biomarkers or cytokine signatures that could predict treatment response would support patient stratification and personalized application. Long-term safety data are also needed, especially regarding repeated exposure of epithelial surfaces to CAP in chronic or oncologic settings, although emerging in vivo evidence on repeated upper-respiratory-tract exposure is beginning to address this question. On the technical front, the miniaturization and standardization of endoscope-compatible CAP devices for use in anatomically complex ORL regions remains a central engineering challenge. Finally, it is imperative to investigate how CAP can be integrated with existing treatment modalities, such as antibiotics, corticosteroids, radiotherapy, or immunotherapies, to achieve synergistic effects.

Addressing these questions through interdisciplinary research and well-designed clinical trials will be essential for establishing CAP as a scientifically grounded, safe, and effective component of future ORL therapeutic strategies.

## Data Availability

No new data were created or analyzed in this study. Data sharing is not applicable to this article.

## References

[1] Weltmann K.D., Kindel E., Woedtke T.v., Hähnel M., Stieber M., Brandenburg R. (2010). Atmospheric-pressure plasma sources: Prospective tools for plasma medicine. Pure Appl. Chem..

[2] Glover J.L., Bendick P.J., Link W.J., Plunkett R.J. (1982). The plasma scalpel: A new thermal knife. Lasers Surg. Med..

[3] Tirakotai W., Mennel H.D., Celik I., Kolodziej M., Bertalanffy H., Riegel T. (2004). Argon plasma coagulation (APC) in brain tumor surgery: Experimental study and clinical experiences. Clin. Neuropathol..

[4] Hoffmann C., Berganza C., Zhang J. (2013). Cold Atmospheric Plasma: Methods of production and application in dentistry and oncology. Med. Gas. Res..

[5] Balzer J., Heuer K., Demir E., Hoffmanns M.A., Baldus S., Fuchs P.C., Awakowicz P., Suschek C.V., Opländer C. (2015). Non-Thermal Dielectric Barrier Discharge (DBD) Effects on Proliferation and Differentiation of Human Fibroblasts Are Primary Mediated by Hydrogen Peroxide. PLoS ONE.

[6] Privat-Maldonado A., Schmidt A., Lin A., Weltmann K.-D., Wende K., Bogaerts A., Bekeschus S. (2019). ROS from Physical Plasmas: Redox Chemistry for Biomedical Therapy. Oxidative Med. Cell. Longev..

[7] Emmert S., van Welzen A., Masur K., Gerling T., Bekeschus S., Eschenburg C., Wahl P., Bernhardt T., Schäfer M., Semmler M.L. (2020). Kaltes Atmosphärendruckplasma zur Behandlung akuter und chronischer Wunden. Der Hautarzt Z. Dermatol. Venerol. Verwandte Geb..

[8] Stratmann B., Costea T.-C., Nolte C., Hiller J., Schmidt J., Reindel J., Masur K., Motz W., Timm J., Kerner W. (2020). Effect of Cold Atmospheric Plasma Therapy vs Standard Therapy Placebo on Wound Healing in Patients With Diabetic Foot Ulcers: A Randomized Clinical Trial. JAMA Netw. Open.

[9] Bakker O., Smits P., van Weersch C., Quaaden M., Bruls E., van Loon A., van der Kleij J. (2025). Improved Wound Healing by Direct Cold Atmospheric Plasma Once or Twice a Week: A Randomized Controlled Trial on Chronic Venous Leg Ulcers. Adv. Wound Care.

[10] Moisan M., Barbeau J., Crevier M.-C., Pelletier J., Philip N., Saoudi B. (2002). Plasma sterilization. Methods and mechanisms. Pure Appl. Chem..

[11] Herve R.C., Kong M.G., Bhatt S., Chen H.L., Comoy E.E., Deslys J.P., Secker T.J., Keevil C.W. (2023). Evaluation of cold atmospheric plasma for the decontamination of flexible endoscopes. J. Hosp. Infect..

[12] Daeschlein G., Napp M., Lutze S., Arnold A., Podewils S.v., Guembel D., Jünger M. (2015). Skin and wound decontamination of multidrug-resistant bacteria by cold atmospheric plasma coagulation. J. Dtsch. Dermatol. Ges. J. Ger. Soc. Dermatol. JDDG.

[13] Maravic T., Petrucci G., Giacomo V.D., Mazzitelli C., Acharya T.R., Kaushik N.K., Choi E.H., Rapino M., D’Urso D., Josic U. (2025). In vitro study of cold atmospheric plasma-induced proliferation inhibition and morphological changes in head and neck carcinoma cell lines. J. Dent..

[14] Kim N., Lee S., Lee S., Kang J., Choi Y.A., Park J., Park C.K., Khang D., Kim S.H. (2022). Portable Cold Atmospheric Plasma Patch-Mediated Skin Anti-Inflammatory Therapy. Adv. Sci..

[15] Canady J., Murthy S.R.K., Zhuang T., Gitelis S., Nissan A., Ly L., Jones O.Z., Cheng X., Adileh M., Blank A.T. (2023). The First Cold Atmospheric Plasma Phase I Clinical Trial for the Treatment of Advanced Solid Tumors: A Novel Treatment Arm for Cancer. Cancers.

[16] Wende K., Bekeschus S., Schmidt A., Jatsch L., Hasse S., Weltmann K.D., Masur K., Woedtke T.v. (2016). Risk assessment of a cold argon plasma jet in respect to its mutagenicity. Mutat. Res. Genet. Toxicol. Environ. Mutagen..

[17] Graves D.B. (2012). The emerging role of reactive oxygen and nitrogen species in redox biology and some implications for plasma applications to medicine and biology. J. Phys. D. Appl. Phys..

[18] Yusupov M., Razzokov J., Cordeiro R.M., Bogaerts A. (2019). Transport of Reactive Oxygen and Nitrogen Species across Aquaporin: A Molecular Level Picture. Oxidative Med. Cell. Longev..

[19] Van der Paal J., Hong S.H., Yusupov M., Gaur N., Oh J.S., Short R.D., Szili E.J., Bogaerts A. (2019). How membrane lipids influence plasma delivery of reactive oxygen species into cells and subsequent DNA damage: An experimental and computational study. Phys. Chem. Chem. Phys..

[20] Yan D., Talbot A., Nourmohammadi N., Sherman J.H., Cheng X., Keidar M. (2015). Toward understanding the selective anticancer capacity of cold atmospheric plasma—A model based on aquaporins (Review). Biointerphases.

[21] Panieri E., Santoro M.M. (2016). ROS homeostasis and metabolism: A dangerous liason in cancer cells. Cell Death Dis..

[22] Cebula M., Schmidt E.E., Arner E.S. (2015). TrxR1 as a potent regulator of the Nrf2-Keap1 response system. Antioxid. Redox Signal.

[23] Liu T., Sun L., Zhang Y., Wang Y., Zheng J. (2022). Imbalanced GSH/ROS and sequential cell death. J. Biochem. Mol. Toxicol..

[24] Sadowska-Bartosz I., Bartosz G. (2023). Peroxiredoxin 2: An Important Element of the Antioxidant Defense of the Erythrocyte. Antioxidants.

[25] Cox A.G., Pearson A.G., Pullar J.M., Jonsson T.J., Lowther W.T., Winterbourn C.C., Hampton M.B. (2009). Mitochondrial peroxiredoxin 3 is more resilient to hyperoxidation than cytoplasmic peroxiredoxins. Biochem. J..

[26] Gorrini C., Harris I.S., Mak T.W. (2013). Modulation of oxidative stress as an anticancer strategy. Nat. Rev. Drug Discov..

[27] Schneider C., Gebhardt L., Arndt S., Karrer S., Zimmermann J.L., Fischer M.J.M., Bosserhoff A.K. (2018). Cold atmospheric plasma causes a calcium influx in melanoma cells triggering CAP-induced senescence. Sci. Rep..

[28] Moniruzzaman R., Rehman M.U., Zhao Q.L., Jawaid P., Mitsuhashi Y., Imaue S., Fujiwara K., Ogawa R., Tomihara K., Saitoh J.I. (2018). Roles of intracellular and extracellular ROS formation in apoptosis induced by cold atmospheric helium plasma and X-irradiation in the presence of sulfasalazine. Free Radic. Biol. Med..

[29] Schuler M., Bossy-Wetzel E., Goldstein J.C., Fitzgerald P., Green D.R. (2000). p53 induces apoptosis by caspase activation through mitochondrial cytochrome c release. J. Biol. Chem..

[30] Semmler M.L., Bekeschus S., Schafer M., Bernhardt T., Fischer T., Witzke K., Seebauer C., Rebl H., Grambow E., Vollmar B. (2020). Molecular Mechanisms of the Efficacy of Cold Atmospheric Pressure Plasma (CAP) in Cancer Treatment. Cancers.

[31] Ayala A., Munoz M.F., Arguelles S. (2014). Lipid peroxidation: Production, metabolism, and signaling mechanisms of malondialdehyde and 4-hydroxy-2-nonenal. Oxidative Med. Cell. Longev..

[32] Fang T., Chen Z., Chen G. (2025). Advances in cold atmospheric plasma therapy for cancer. Bioact. Mater..

[33] Seiler A., Schneider M., Forster H., Roth S., Wirth E.K., Culmsee C., Plesnila N., Kremmer E., Radmark O., Wurst W. (2008). Glutathione peroxidase 4 senses and translates oxidative stress into 12/15-lipoxygenase dependent- and AIF-mediated cell death. Cell Metab..

[34] Bekeschus S., Eisenmann S., Sagwal S.K., Bodnar Y., Moritz J., Poschkamp B., Stoffels I., Emmert S., Madesh M., Weltmann K.-D. (2020). xCT (SLC7A11) expression confers intrinsic resistance to physical plasma treatment in tumor cells. Redox Biol..

[35] Schmidt A., Bekeschus S., Jarick K., Hasse S., von Woedtke T., Wende K. (2019). Cold Physical Plasma Modulates p53 and Mitogen-Activated Protein Kinase Signaling in Keratinocytes. Oxidative Med. Cell. Longev..

[36] Motaln H., Recek N., Rogelj B. (2021). Intracellular Responses Triggered by Cold Atmospheric Plasma and Plasma-Activated Media in Cancer Cells. Molecules.

[37] Yan D., Wang Q., Adhikari M., Malyavko A., Lin L., Zolotukhin D.B., Yao X., Kirschner M., Sherman J.H., Keidar M. (2020). A Physically Triggered Cell Death via Transbarrier Cold Atmospheric Plasma Cancer Treatment. ACS Appl. Mater. Interfaces.

[38] Bauer G. (2019). The synergistic effect between hydrogen peroxide and nitrite, two long-lived molecular species from cold atmospheric plasma, triggers tumor cells to induce their own cell death. Redox Biol..

[39] Mizuno K., Yonetamari K., Shirakawa Y., Akiyama T., Ono R. (2017). Anti-tumor immune response induced by nanosecond pulsed streamer discharge in mice. J. Phys. D. Appl. Phys..

[40] Faramarzi F., Zafari P., Alimohammadi M., Moonesi M., Rafiei A., Bekeschus S. (2021). Cold Physical Plasma in Cancer Therapy: Mechanisms, Signaling, and Immunity. Oxidative Med. Cell. Longev..

[41] Azzariti A., Iacobazzi R.M., Di Fonte R., Porcelli L., Gristina R., Favia P., Fracassi F., Trizio I., Silvestris N., Guida G. (2019). Plasma-activated medium triggers cell death and the presentation of immune activating danger signals in melanoma and pancreatic cancer cells. Sci. Rep..

[42] Yan D., Talbot A., Nourmohammadi N., Cheng X., Canady J., Sherman J., Keidar M. (2015). Principles of using Cold Atmospheric Plasma Stimulated Media for Cancer Treatment. Sci. Rep..

[43] Laroussi M., Lu X. (2005). Room-temperature atmospheric pressure plasma plume for biomedical applications. Appl. Phys. Lett..

[44] Kuchenbecker M., Bibinov N., Kaemlimg A., Wandke D., Awakowicz P., Viöl W. (2009). Characterization of DBD plasma source for biomedical applications. J. Phys. D. Appl. Phys..

[45] Ploenes L., Haas D., Zhang D., van de Meerakker S.Y.T., Willitsch S. (2016). Cold and intense OH radical beam sources. Rev. Sci. Instrum..

[46] Napp J., Daeschlein G., Napp M., Podewils S.v., Gümbel D., Spitzmueller R., Fornaciari P., Hinz P., Jünger M. (2015). On the history of plasma treatment and comparison of microbiostatic efficacy of a historical high-frequency plasma device with two modern devices. GMS Hyg. Infect. Control.

[47] Yan D., Sherman J.H., Keidar M. (2017). Cold atmospheric plasma, a novel promising anti-cancer treatment modality. Oncotarget.

[48] Liu D.X., Liu Z.C., Chen C., Yang A.J., Li D., Rong M.Z., Chen H.L., Kong M.G. (2016). Aqueous reactive species induced by a surface air discharge: Heterogeneous mass transfer and liquid chemistry pathways. Sci. Rep..

[49] Jha N., Ryu J.J., Choi E.H., Kaushik N.K. (2017). Generation and Role of Reactive Oxygen and Nitrogen Species Induced by Plasma, Lasers, Chemical Agents, and Other Systems in Dentistry. Oxidative Med. Cell. Longev..

[50] Kaushik N.K., Ghimire B., Li Y., Adhikari M., Veerana M., Kaushik N., Jha N., Adhikari B., Lee S.-J., Masur K. (2018). Biological and medical applications of plasma-activated media, water and solutions. Biol. Chem..

[51] Tsoukou E., Bourke P., Boehm D. (2020). Temperature Stability and Effectiveness of Plasma-Activated Liquids over an 18 Months Period. Water.

[52] Dubuc A., Monsarrat P., Virard F., Merbahi N., Sarrette J.P., Laurencin-Dalicieux S., Cousty S. (2018). Use of cold-atmospheric plasma in oncology: A concise systematic review. Ther. Adv. Med. Oncol..

[53] Sen C.K. (2009). Wound healing essentials: Let there be oxygen. Wound Repair Regen..

[54] Morton L.M., Phillips T.J. (2016). Wound healing and treating wounds: Differential diagnosis and evaluation of chronic wounds. J. Am. Acad. Dermatol..

[55] Dunnill C., Patton T., Brennan J., Barrett J., Dryden M., Cooke J., Leaper D., Georgopoulos N.T. (2017). Reactive oxygen species (ROS) and wound healing: The functional role of ROS and emerging ROS-modulating technologies for augmentation of the healing process. Int. Wound J..

[56] Marches A., Clement E., Albérola G., Rols M.-P., Cousty S., Simon M., Merbahi N. (2022). Cold Atmospheric Plasma Jet Treatment Improves Human Keratinocyte Migration and Wound Closure Capacity without Causing Cellular Oxidative Stress. Int. J. Mol. Sci..

[57] Hiller J., Stratmann B., Timm J., Costea T.-C., Tschoepe D. (2022). Enhanced growth factor expression in chronic diabetic wounds treated by cold atmospheric plasma. Diabet. Med. A J. Br. Diabet. Assoc..

[58] Frykberg R.G., Banks J. (2015). Challenges in the Treatment of Chronic Wounds. Adv. Wound Care.

[59] Kolimi P., Narala S., Nyavanandi D., Youssef A.A.A., Dudhipala N. (2022). Innovative Treatment Strategies to Accelerate Wound Healing: Trajectory and Recent Advancements. Cells.

[60] Lohuis P.J.F.M., Maldonado-Chapa F., Santos-Santillana K.M., Filipović B., Dirven R., Karakullukcku M.B., Karssemakers L., Schreuder W.H., Zuur C.L., Timmermans J. (2023). Optimizing Wound Care after Surgery of the Head and Neck: A Review of Dressing Materials. Facial Plast. Surg. FPS.

[61] Xue M., Jackson C.J. (2015). Extracellular Matrix Reorganization During Wound Healing and Its Impact on Abnormal Scarring. Adv. Wound Care.

[62] Kisch T., Helmke A., Schleusser S., Song J., Liodaki E., Stang F.H., Mailaender P., Kraemer R. (2016). Improvement of cutaneous microcirculation by cold atmospheric plasma (CAP): Results of a controlled, prospective cohort study. Microvasc. Res..

[63] Bekeschus S., Schmidt A., Bethge L., Masur K., Woedtke T.v., Hasse S., Wende K. (2016). Redox Stimulation of Human THP-1 Monocytes in Response to Cold Physical Plasma. Oxidative Med. Cell. Longev..

[64] Haertel B., Woedtke T.v., Weltmann K.-D., Lindequist U. (2014). Non-thermal atmospheric-pressure plasma possible application in wound healing. Biomol. Ther..

[65] Pastar I., Stojadinovic O., Yin N.C., Ramirez H., Nusbaum A.G., Sawaya A., Patel S.B., Khalid L., Isseroff R.R., Tomic-Canic M. (2014). Epithelialization in Wound Healing: A Comprehensive Review. Adv. Wound Care.

[66] Weltmann K.-D., von Woedtke T. (2017). Plasma medicine—Current state of research and medical application. Plasma Phys. Control. Fusion.

[67] Blackert S., Haertel B., Wende K., Woedtke T.v., Lindequist U. (2013). Influence of non-thermal atmospheric pressure plasma on cellular structures and processes in human keratinocytes (HaCaT). J. Dermatol. Sci..

[68] Hasse S., Duong Tran T., Hahn O., Kindler S., Metelmann H.-R., Woedtke T.v., Masur K. (2016). Induction of proliferation of basal epidermal keratinocytes by cold atmospheric-pressure plasma. Clin. Exp. Dermatol..

[69] Arndt S., Schmidt A., Karrer S., Woedtke T.v. (2018). Comparing two different plasma devices kINPen and Adtec SteriPlas regarding their molecular and cellular effects on wound healing. Clin. Plasma Med..

[70] Schmidt A., Bekeschus S., Wende K., Vollmar B., Woedtke T.v. (2017). A cold plasma jet accelerates wound healing in a murine model of full-thickness skin wounds. Exp. Dermatol..

[71] Dzimitrowicz A., Bielawska-Pohl A., Jamroz P., Dora J., Krawczenko A., Busco G., Grillon C., Kieda C., Klimczak A., Terefinko D. (2020). Activation of the Normal Human Skin Cells by a Portable Dielectric Barrier Discharge-Based Reaction-Discharge System of a Defined Gas Temperature. Plasma Chem. Plasma Process..

[72] Schmidt A., Bekeschus S. (2018). Redox for Repair: Cold Physical Plasmas and Nrf2 Signaling Promoting Wound Healing. Antioxidants.

[73] Schmidt A., Woedtke T.v., Weltmann K.-D., Masur K. (2013). Identification of the Molecular Basis of Non-thermal Plasma-Induced Changes in Human Keratinocytes. Plasma Med..

[74] Arndt S., Unger P., Wacker E., Shimizu T., Heinlin J., Li Y.-F., Thomas H.M., Morfill G.E., Zimmermann J.L., Bosserhoff A.-K. (2013). Cold atmospheric plasma (CAP) changes gene expression of key molecules of the wound healing machinery and improves wound healing in vitro and in vivo. PLoS ONE.

[75] García-Alcantara E., López-Callejas R., Morales-Ramírez P.R., Peña-Eguiluz R., Fajardo-Muñoz R., Mercado-Cabrera A., Barocio S.R., Valencia-Alvarado R., Rodríguez-Méndez B.G., Muñoz-Castro A.E. (2013). Accelerated mice skin acute wound healing in vivo by combined treatment of argon and helium plasma needle. Arch. Med. Res..

[76] Nasruddin, Nakajima Y., Mukai K., Rahayu H.S.E., Nur M., Ishijima T., Enomoto H., Uesugi Y., Sugama J., Nakatani T. (2014). Cold plasma on full-thickness cutaneous wound accelerates healing through promoting inflammation, re-epithelialization and wound contraction. Clin. Plasma Med..

[77] Xu G.-M., Shi X.-M., Cai J.-F., Chen S.-L., Li P., Yao C.-W., Chang Z.-S., Zhang G.-J. (2015). Dual effects of atmospheric pressure plasma jet on skin wound healing of mice. Wound Repair Regen..

[78] Ulrich C., Kluschke F., Patzelt A., Vandersee S., Czaika V.A., Richter H., Bob A., Hutten J.v., Painsi C., Hüge R. (2015). Clinical use of cold atmospheric pressure argon plasma in chronic leg ulcers: A pilot study. J. Wound Care.

[79] Hartwig S., Doll C., Voss J.O., Hertel M., Preissner S., Raguse J.D. (2017). Treatment of Wound Healing Disorders of Radial Forearm Free Flap Donor Sites Using Cold Atmospheric Plasma: A Proof of Concept. J. Oral Maxillofac. Surg. Off. J. Am. Assoc. Oral Maxillofac. Surg..

[80] Raissi-Dehkordi N., Raissi-Dehkordi N., Ebrahimibagha H., Tayebi T., Moeinabadi-Bidgoli K., Hassani M., Niknejad H. (2025). Advancing chronic and acute wound healing with cold atmospheric plasma: Cellular and molecular mechanisms, benefits, risks, and future directions. Front. Med..

[81] Woedtke T.v., Emmert S., Metelmann H.-R., Rupf S., Weltmann K.-D. (2020). Perspectives on cold atmospheric plasma (CAP) applications in medicine. Phys. Plasmas.

[82] Izadjoo M., Zack S., Kim H., Skiba J. (2018). Medical applications of cold atmospheric plasma: State of the science. J. Wound Care.

[83] Ziuzina D., Boehm D., Patil S., Cullen P.J., Bourke P. (2015). Cold Plasma Inactivation of Bacterial Biofilms and Reduction of Quorum Sensing Regulated Virulence Factors. PLoS ONE.

[84] Sun P., Sun Y., Wu H., Zhu W., Lopez J.L., Liu W., Zhang J., Li R., Fang J. (2011). Atmospheric pressure cold plasma as an antifungal therapy. Appl. Phys. Lett..

[85] Mai-Prochnow A., Bradbury M., Ostrikov K., Murphy A.B. (2015). Pseudomonas aeruginosa Biofilm Response and Resistance to Cold Atmospheric Pressure Plasma Is Linked to the Redox-Active Molecule Phenazine. PLoS ONE.

[86] Napp M., Daeschlein G., Podewils S.v., Hinz P., Emmert S., Haase H., Spitzmueller R., Gümbel D., Kasch R., Jünger M. (2016). In vitro susceptibility of methicillin-resistant and methicillin-susceptible strains of Staphylococcus aureus to two different cold atmospheric plasma sources. Infection.

[87] Mohd Nasir N., Lee B.K., Yap S.S., Thong K.L., Yap S.L. (2016). Cold plasma inactivation of chronic wound bacteria. Arch. Biochem. Biophys..

[88] Lunder M., Dahle S., Fink R. (2024). Cold atmospheric plasma for surface disinfection: A promising weapon against deleterious meticillin-resistant Staphylococcus aureus biofilms. J. Hosp. Infect..

[89] Nicol M.J., Brubaker T.R., Honish B.J., Simmons A.N., Kazemi A., Geissel M.A., Whalen C.T., Siedlecki C.A., Bilén S.G., Knecht S.D. (2020). Antibacterial effects of low-temperature plasma generated by atmospheric-pressure plasma jet are mediated by reactive oxygen species. Sci. Rep..

[90] Boekema B., Stoop M., Vlig M., van Liempt J., Sobota A., Ulrich M., Middelkoop E. (2021). Antibacterial and safety tests of a flexible cold atmospheric plasma device for the stimulation of wound healing. Appl. Microbiol. Biotechnol..

[91] Ding R., Song J., Huang X., Tan L., Rao X., Yang Y. (2025). Treatment of Clinically Important Bacteria With Cold Atmospheric Plasma. Microb. Biotechnol..

[92] Heisterberg L., Biber U., Neugebauer A., Liese J., Thompson C.C., Enderle M., Grashorn S. (2026). Argon plasma-activated liquid inactivates Helicobacter pylori and resistant hospital pathogens through acidification and reactive species. Front. Microbiol..

[93] Kehrmann J., Koch F., Zumdick S., Howner A., Best L., Masshofer L., Scharfenberg S., Zeschnigk M., Becker J.C., Schadendorf D. (2023). Reduced Staphylococcus Abundance Characterizes the Lesional Microbiome of Actinic Keratosis Patients after Field-Directed Therapies. Microbiol. Spectr..

[94] Brugger S.D., Bomar L., Lemon K.P. (2016). Commensal-Pathogen Interactions along the Human Nasal Passages. PLoS Pathog..

[95] Rawlings B.A., Higgins T.S., Han J.K. (2013). Bacterial pathogens in the nasopharynx, nasal cavity, and osteomeatal complex during wellness and viral infection. Am. J. Rhinol. Allergy.

[96] Gelardi M., Giancaspro R., Cassano M. (2023). Biofilm in sino-nasal infectious diseases: The role nasal cytology in the diagnostic work up and therapeutic implications. Eur. Arch. Oto-Rhino-Laryngol..

[97] Vickery T.W., Ramakrishnan V.R., Suh J.D. (2019). The Role of Staphylococcus aureus in Patients with Chronic Sinusitis and Nasal Polyposis. Curr. Allergy Asthma Rep..

[98] Rowan N.R., Lee S., Sahu N., Kanaan A., Cox S., Phillips C.D., Wang E.W. (2015). The role of viruses in the clinical presentation of chronic rhinosinusitis. Am. J. Rhinol. Allergy.

[99] Cho G.S., Moon B.-J., Lee B.-J., Gong C.-H., Kim N.H., Kim Y.-S., Kim H.S., Jang Y.J. (2013). High rates of detection of respiratory viruses in the nasal washes and mucosae of patients with chronic rhinosinusitis. J. Clin. Microbiol..

[100] Haque M., McKimm J., Godman B., Abu Bakar M., Sartelli M. (2019). Initiatives to reduce postoperative surgical site infections of the head and neck cancer surgery with a special emphasis on developing countries. Expert Rev. Anticancer Ther..

[101] Lindsay S., Oates A., Bourdillon K. (2017). The detrimental impact of extracellular bacterial proteases on wound healing. Int. Wound J..

[102] Krysko O., Teufelberger A., Van Nevel S., Krysko D.V., Bachert C. (2019). Protease/antiprotease network in allergy: The role of Staphylococcus aureus protease-like proteins. Allergy.

[103] Hong H., Liao S., Chen F., Yang Q., Wang D.Y. (2020). Role of IL-25, IL-33, and TSLP in triggering united airway diseases toward type 2 inflammation. Allergy.

[104] Teufelberger A.R., Van Nevel S., Hulpiau P., Nordengrun M., Savvides S.N., De Graeve S., Akula S., Holtappels G., De Ruyck N., Declercq W. (2020). Mouse Strain-Dependent Difference Toward the Staphylococcus aureus Allergen Serine Protease-Like Protein D Reveals a Novel Regulator of IL-33. Front. Immunol..

[105] Emicke P. (2012). Proteome Dynamics of non-Thermal Atmospheric Plasma Treated Airway Epithelial Cells. Ph.D. Thesis.

[106] Dubey S.K., Parab S., Alexander A., Agrawal M., Achalla V.P.K., Pal U.N., Pandey M.M., Kesharwani P. (2022). Cold atmospheric plasma therapy in wound healing. Process Biochem..

[107] Rasouli M., Fallah N., Bekeschus S. (2021). Combining Nanotechnology and Gas Plasma as an Emerging Platform for Cancer Therapy: Mechanism and Therapeutic Implication. Oxidative Med. Cell. Longev..

[108] Abdo A.I., Nguyen H.T., Kaul L.D., Schmitt-John T., Kopecki Z., Ogunniyi A.D., Richter K. (2025). Plasma-activated water accelerates wound healing and reduces Staphylococcus aureus infection in vivo. Biofilm.

[109] Fridman W.H., Pagès F., Sautès-Fridman C., Galon J. (2012). The immune contexture in human tumours: Impact on clinical outcome. Nat. Rev. Cancer.

[110] Kaushik N.K., Kaushik N., Min B., Choi K.H., Hong Y.J., Miller V., Fridman A., Choi E.H. (2016). Cytotoxic macrophage-released tumour necrosis factor-alpha (TNF-α) as a killing mechanism for cancer cell death after cold plasma activation. J. Phys. D. Appl. Phys..

[111] Kaushik N.K., Kaushik N., Adhikari M., Ghimire B., Linh N.N., Mishra Y.K., Lee S.-J., Choi E.H. (2019). Preventing the Solid Cancer Progression via Release of Anticancer-Cytokines in Co-Culture with Cold Plasma-Stimulated Macrophages. Cancers.

[112] Zivanic M., Espona-Noguera A., Lin A., Canal C. (2023). Current State of Cold Atmospheric Plasma and Cancer-Immunity Cycle: Therapeutic Relevance and Overcoming Clinical Limitations Using Hydrogels. Adv. Sci..

[113] Tran Janco J.M., Lamichhane P., Karyampudi L., Knutson K.L. (2015). Tumor-infiltrating dendritic cells in cancer pathogenesis. J. Immunol..

[114] Michielsen A.J., Hogan A.E., Marry J., Tosetto M., Cox F., Hyland J.M., Sheahan K.D., O’Donoghue D.P., Mulcahy H.E., Ryan E.J. (2011). Tumour tissue microenvironment can inhibit dendritic cell maturation in colorectal cancer. PLoS ONE.

[115] van Loenhout J., Flieswasser T., Freire Boullosa L., Waele J.d., van Audenaerde J., Marcq E., Jacobs J., Lin A., Lion E., Dewitte H. (2019). Cold Atmospheric Plasma-Treated PBS Eliminates Immunosuppressive Pancreatic Stellate Cells and Induces Immunogenic Cell Death of Pancreatic Cancer Cells. Cancers.

[116] Vitiello L., Gorini S., Rosano G., La Sala A. (2012). Immunoregulation through extracellular nucleotides. Blood.

[117] Wang Y., Abazid A., Badendieck S., Mustea A., Stope M.B. (2023). Impact of Non-Invasive Physical Plasma on Heat Shock Protein Functionality in Eukaryotic Cells. Biomedicines.

[118] La Sala A., Ferrari D., Di Virgilio F., Idzko M., Norgauer J., Girolomoni G. (2003). Alerting and tuning the immune response by extracellular nucleotides. J. Leukoc. Biol..

[119] Wolff C.M., Kolb J.F., Bekeschus S. (2022). Combined In Vitro Toxicity and Immunogenicity of Cold Plasma and Pulsed Electric Fields. Biomedicines.

[120] Arndt S., Landthaler M., Zimmermann J.L., Unger P., Wacker E., Shimizu T., Li Y.F., Morfill G.E., Bosserhoff A.K., Karrer S. (2015). Effects of cold atmospheric plasma (CAP) on ß-defensins, inflammatory cytokines, and apoptosis-related molecules in keratinocytes in vitro and in vivo. PLoS ONE.

[121] Li X., Rui X., Li D., Wang Y., Tan F. (2022). Plasma oncology: Adjuvant therapy for head and neck cancer using cold atmospheric plasma. Front. Oncol..

[122] Ngwa W., Irabor O.C., Schoenfeld J.D., Hesser J., Demaria S., Formenti S.C. (2018). Using immunotherapy to boost the abscopal effect. Nat. Rev. Cancer.

[123] Park J., Jang Y.-S., Choi J.-H., Ryu M., Kim G.-C., Byun J.-H., Hwang D.-S., Kim U.-K. (2021). Anticancer Efficacy of Nonthermal Plasma Therapy Combined with PD-L1 Antibody Conjugated Gold Nanoparticles on Oral Squamous Cell Carcinoma. Appl. Sci..

[124] Chen G., Chen Z., Wen D., Wang Z., Li H., Zeng Y., Dotti G., Wirz R.E., Gu Z. (2020). Transdermal cold atmospheric plasma-mediated immune checkpoint blockade therapy. Proc. Natl. Acad. Sci. USA.

[125] Wang Y., Mang X., Li D., Wang Z., Chen Y., Cai Z., Tan F. (2024). Cold atmospheric plasma sensitizes head and neck cancer to chemotherapy and immune checkpoint blockade therapy. Redox Biol..

[126] Lendeckel D., Eymann C., Emicke P., Daeschlein G., Darm K., O’Neil S., Beule A.G., von Woedtke T., Volker U., Weltmann K.D. (2015). Proteomic Changes of Tissue-Tolerable Plasma Treated Airway Epithelial Cells and Their Relation to Wound Healing. BioMed Res. Int..

[127] Pavlovic O., Lazarevic M., Jakovljevic A., Skoro N., Puac N., Mojsilovic S., Miletic M. (2025). Antitumor Potential of Different Treatment Approaches Using Cold Atmospheric Pressure Plasma on Oral Squamous Cell Carcinoma Models: In Vitro Study. Biomedicines.

[128] Kim S.J., Chung T.H. (2016). Cold atmospheric plasma jet-generated RONS and their selective effects on normal and carcinoma cells. Sci. Rep..

[129] Schlegel J., Köritzer J., Boxhammer V. (2013). Plasma in cancer treatment. Clin. Plasma Med..

[130] Metelmann H.-R., Seebauer C., Miller V., Fridman A., Bauer G., Graves D.B., Pouvesle J.-M., Rutkowski R., Schuster M., Bekeschus S. (2018). Clinical experience with cold plasma in the treatment of locally advanced head and neck cancer. Clin. Plasma Med..

[131] Gay-Mimbrera J., García M.C., Isla-Tejera B., Rodero-Serrano A., García-Nieto A.V., Ruano J. (2016). Clinical and Biological Principles of Cold Atmospheric Plasma Application in Skin Cancer. Adv. Ther..

[132] Dai X., Bazaka K., Thompson E.W., Ostrikov K.K. (2020). Cold Atmospheric Plasma: A Promising Controller of Cancer Cell States. Cancers.

[133] Liu H., Liu X., Zhang C., Zhu H., Xu Q., Bu Y., Lei Y. (2017). Redox Imbalance in the Development of Colorectal Cancer. J. Cancer.

[134] Dequanter D., Dok R., Nuyts S. (2017). Basal oxidative stress ratio of head and neck squamous cell carcinomas correlates with nodal metastatic spread in patients under therapy. OncoTargets Ther..

[135] Hirst A.M., Simms M.S., Mann V.M., Maitland N.J., O’Connell D., Frame F.M. (2015). Low-temperature plasma treatment induces DNA damage leading to necrotic cell death in primary prostate epithelial cells. Br. J. Cancer.

[136] Kaushik N.K., Kaushik N., Park D., Choi E.H. (2014). Altered antioxidant system stimulates dielectric barrier discharge plasma-induced cell death for solid tumor cell treatment. PLoS ONE.

[137] Walk R.M., Snyder J.A., Srinivasan P., Kirsch J., Diaz S.O., Blanco F.C., Shashurin A., Keidar M., Sandler A.D. (2013). Cold atmospheric plasma for the ablative treatment of neuroblastoma. J. Pediatr. Surg..

[138] Kater A.P., Seymour J.F., Hillmen P., Eichhorst B., Langerak A.W., Owen C., Verdugo M., Wu J., Punnoose E.A., Jiang Y. (2019). Fixed Duration of Venetoclax-Rituximab in Relapsed/Refractory Chronic Lymphocytic Leukemia Eradicates Minimal Residual Disease and Prolongs Survival: Post-Treatment Follow-Up of the MURANO Phase III Study. J. Clin. Oncol. Off. J. Am. Soc. Clin. Oncol..

[139] Seymour J.F., Mobasher M., Kater A.P. (2018). Venetoclax-Rituximab in Chronic Lymphocytic Leukemia. N. Engl. J. Med..

[140] Welz C., Emmert S., Canis M., Becker S., Baumeister P., Shimizu T., Morfill G.E., Harréus U., Zimmermann J.L. (2015). Cold Atmospheric Plasma: A Promising Complementary Therapy for Squamous Head and Neck Cancer. PLoS ONE.

[141] Guerrero-Preston R., Ogawa T., Uemura M., Shumulinsky G., Valle B.L., Pirini F., Ravi R., Sidransky D., Keidar M., Trink B. (2014). Cold atmospheric plasma treatment selectively targets head and neck squamous cell carcinoma cells. Int. J. Mol. Med..

[142] Chang J.W., Kang S.U., Shin Y.S., Kim K.I., Seo S.J., Yang S.S., Lee J.-S., Moon E., Baek S.J., Lee K. (2014). Non-thermal atmospheric pressure plasma induces apoptosis in oral cavity squamous cell carcinoma: Involvement of DNA-damage-triggering sub-G(1) arrest via the ATM/p53 pathway. Arch. Biochem. Biophys..

[143] Chang J.W., Kang S.U., Shin Y.S., Seo S.J., Kim Y.S., Yang S.S., Lee J.-S., Moon E., Lee K., Kim C.-H. (2015). Combination of NTP with cetuximab inhibited invasion/migration of cetuximab-resistant OSCC cells: Involvement of NF-κB signaling. Sci. Rep..

[144] Brunner T.F., Probst F.A., Troeltzsch M., Schwenk-Zieger S., Zimmermann J.L., Morfill G., Becker S., Harréus U., Welz C. (2022). Primary cold atmospheric plasma combined with low dose cisplatin as a possible adjuvant combination therapy for HNSCC cells-an in-vitro study. Head. Face Med..

[145] Sklias K., Santos Sousa J., Girard P.M. (2021). Role of Short- and Long-Lived Reactive Species on the Selectivity and Anti-Cancer Action of Plasma Treatment In Vitro. Cancers.

[146] Manzhula K., Rebl A., Budde-Sagert K., Rebl H. (2024). Interplay of Cellular Nrf2/NF-kappaB Signalling after Plasma Stimulation of Malignant vs. Non-Malignant Dermal Cells. Int. J. Mol. Sci..

[147] Yan D., Malyavko A., Wang Q., Ostrikov K.K., Sherman J.H., Keidar M. (2021). Multi-Modal Biological Destruction by Cold Atmospheric Plasma: Capability and Mechanism. Biomedicines.

[148] Braný D., Dvorská D., Strnádel J., Matáková T., Halašová E., Škovierová H. (2021). Effect of Cold Atmospheric Plasma on Epigenetic Changes, DNA Damage, and Possibilities for Its Use in Synergistic Cancer Therapy. Int. J. Mol. Sci..

[149] Dai X., Thompson E.W., Ostrikov K.K. (2022). Receptor-Mediated Redox Imbalance: An Emerging Clinical Avenue against Aggressive Cancers. Biomolecules.

[150] Perrotti V., Caponio V.C.A., Muzio L.L., Choi E.H., Di Marcantonio M.C., Mazzone M., Kaushik N.K., Mincione G. (2022). Open Questions in Cold Atmospheric Plasma Treatment in Head and Neck Cancer: A Systematic Review. Int. J. Mol. Sci..

[151] Sathe N.C., Zito P.M. (2025). Skin Cancer. StatPearls.

[152] Bekeschus S., Singer D., Ratnayake G., Ruhnau K., Ostrikov K., Thompson E.W. (2025). Rationales of Cold Plasma Jet Therapy in Skin Cancer. Exp. Dermatol..

[153] Pavri S.N., Clune J., Ariyan S., Narayan D. (2016). Malignant Melanoma: Beyond the Basics. Plast. Reconstr. Surg..

[154] Arndt S., Wacker E., Li Y.-F., Shimizu T., Thomas H.M., Morfill G.E., Karrer S., Zimmermann J.L., Bosserhoff A.-K. (2013). Cold atmospheric plasma, a new strategy to induce senescence in melanoma cells. Exp. Dermatol..

[155] Schmidt A., Bekeschus S., Woedtke T.v., Hasse S. (2015). Cell migration and adhesion of a human melanoma cell line is decreased by cold plasma treatment. Clin. Plasma Med..

[156] Chang J.W., Kang S.U., Shin Y.S., Kim K.I., Seo S.J., Yang S.S., Lee J.-S., Moon E., Lee K., Kim C.-H. (2014). Non-thermal atmospheric pressure plasma inhibits thyroid papillary cancer cell invasion via cytoskeletal modulation, altered MMP-2/-9/uPA activity. PLoS ONE.

[157] Alimohammadi M., Golpur M., Sohbatzadeh F., Hadavi S., Bekeschus S., Niaki H.A., Valadan R., Rafiei A. (2020). Cold Atmospheric Plasma Is a Potent Tool to Improve Chemotherapy in Melanoma In Vitro and In Vivo. Biomolecules.

[158] Adhikari M., Adhikari B., Ghimire B., Baboota S., Choi E.H. (2020). Cold Atmospheric Plasma and Silymarin Nanoemulsion Activate Autophagy in Human Melanoma Cells. Int. J. Mol. Sci..

[159] Tan F., Wang Y., Zhang S., Shui R., Chen J. (2022). Plasma Dermatology: Skin Therapy Using Cold Atmospheric Plasma. Front. Oncol..

[160] Kordt M., Trautmann I., Schlie C., Lindner T., Stenzel J., Schildt A., Boeckmann L., Bekeschus S., Kurth J., Krause B.J. (2021). Multimodal Imaging Techniques to Evaluate the Anticancer Effect of Cold Atmospheric Pressure Plasma. Cancers.

[161] Volotskova O., Hawley T.S., Stepp M.A., Keidar M. (2012). Targeting the cancer cell cycle by cold atmospheric plasma. Sci. Rep..

[162] Wang L., Yang X., Yang C., Gao J., Zhao Y., Cheng C., Zhao G., Liu S. (2019). The inhibition effect of cold atmospheric plasma-activated media in cutaneous squamous carcinoma cells. Future Oncol..

[163] van der Paal J., Neyts E.C., Verlackt C.C.W., Bogaerts A. (2016). Effect of lipid peroxidation on membrane permeability of cancer and normal cells subjected to oxidative stress. Chem. Sci..

[164] Shi L., Yu L., Zou F., Hu H., Liu K., Lin Z. (2017). Gene expression profiling and functional analysis reveals that p53 pathway-related gene expression is highly activated in cancer cells treated by cold atmospheric plasma-activated medium. PeerJ.

[165] Choi J.H., Gu H.J., Park K.H., Hwang D.S., Kim G.C. (2022). Anti-Cancer Activity of the Combinational Treatment of Noozone Cold Plasma with p-FAK Antibody-Conjugated Gold Nanoparticles in OSCC Xenograft Mice. Biomedicines.

[166] Qi W., Liu H., Liu H., Guo Y., Wu L., Bao C., Liu X. (2025). Synergistical Induction of Apoptosis via Cold Atmospheric Plasma and Nanohydroxyapatite for Selective Inhibition of Oral Squamous Cell Carcinoma in Tumour Microenvironment. Cell Prolif..

[167] Cheng Y.C., Chang K.W., Pan J.H., Chen C.Y., Chou C.H., Tu H.F., Li W.C., Lin S.C. (2023). Cold Atmospheric Plasma Jet Irradiation Decreases the Survival and the Expression of Oncogenic miRNAs of Oral Carcinoma Cells. Int. J. Mol. Sci..

[168] Jang H., Jang Y.S., Kim G.C., Hwang D.S. (2025). Plasma-activated medium induces apoptosis of oral squamous cell carcinoma through the MAPKs pathway. Sci. Rep..

[169] Holanda A.G.A., Cesario B.C., Silva V.M., Francelino L.E.C., Nascimento B.H.M., Damasceno K.F.A., Ishikawa U., Farias N.B.S., Junior R.F.A., Barboza C.A.G. (2023). Use of Cold Atmospheric Plasma in the Treatment of Squamous Cell Carcinoma: In Vitro Effects and Clinical Application in Feline Tumors: A Pilot Study. Top. Companion Anim. Med..

[170] di Giacomo V., Balaha M., Pece A., Cela I., Fulgenzi G., Orsini G., Spadoni T., Acharya T.R., Kaushik N.K., Choi E.H. (2025). Human head and neck cancer cell lines response to cold atmospheric plasma activated media is affected by the chemistry of culture media. Heliyon.

[171] di Giacomo V., Balaha M., Pinti M., Di Marcantonio M.C., Cela I., Acharya T.R., Kaushik N.K., Choi E.H., Mincione G., Sala G. (2025). Cold atmospheric plasma activated media selectively affects human head and neck cancer cell lines. Oral Dis..

[172] Seebauer C., Freund E., Dieke T., Hasse S., Segebarth M., Rautenberg C., Metelmann H.R., Bekeschus S. (2023). Decreased inflammatory profile in oral leukoplakia tissue exposed to cold physical plasma ex vivo. J. Oral Pathol. Med..

[173] Jung S.-N., Oh C., Chang J.W., Liu L., Lim M.A., Jin Y.L., Piao Y., Kim H.J., Won H.-R., Lee S.E. (2021). EGR1/GADD45α Activation by ROS of Non-Thermal Plasma Mediates Cell Death in Thyroid Carcinoma. Cancers.

[174] Wang P., Zhou R., Zhou R., Li W., Weerasinghe J., Chen S., Rehm B.H.A., Zhao L., Frentiu F.D., Zhang Z. (2022). Cold atmospheric plasma for preventing infection of viruses that use ACE2 for entry. Theranostics.

[175] Mrochen D.M., Miebach L., Skowski H., Bansemer R., Drechsler C.A., Hofmanna U., Hein M., Mamat U., Gerling T., Schaible U. (2022). Toxicity and virucidal activity of a neon-driven micro plasma jet on eukaryotic cells and a coronavirus. Free Radic. Biol. Med..

[176] Won H.R., Kang S.U., Kim H.J., Jang J.Y., Shin Y.S., Kim C.H. (2018). Non-thermal plasma treated solution with potential as a novel therapeutic agent for nasal mucosa regeneration. Sci. Rep..

[177] Oguzlu H., Baldelli A., Mohammadi X., Kong A., Bacca M., Pratap-Singh A. (2024). Cold Plasma for the Modification of the Surface Roughness of Microparticles. ACS Omega.

[178] Adan A.A., Jelle N. (2025). The use of cold atmospheric plasma/J-Plasma in the management of rhinophyma: A case report. JPRAS Open.

[179] Karrer S., Unger P., Gruber M., Gebhardt L., Schober R., Berneburg M., Bosserhoff A.K., Arndt S. (2024). In Vitro Safety Study on the Use of Cold Atmospheric Plasma in the Upper Respiratory Tract. Cells.

[180] Reichold L.Z., Gruber M., Unger P., Maisch T., Lindner R., Gebhardt L., Schober R., Karrer S., Arndt S. (2024). Cellular Response of Immune Cells in the Upper Respiratory Tract After Treatment with Cold Atmospheric Plasma In Vitro. Int. J. Mol. Sci..

[181] Arndt S., Unger P., Gebhardt L., Schober R., Berneburg M., Karrer S. (2025). In Vivo Immune Cell Responses and Long-Term Effects of Cold Atmospheric Plasma in the Upper Respiratory Tract. Int. J. Mol. Sci..

[182] Robert E., Vandamme M., Brullé L., Lerondel S., Le Pape A., Sarron V., Riés D., Darny T., Dozias S., Collet G. (2013). Perspectives of endoscopic plasma applications. Clin. Plasma Med..

[183] Scharf C. (2022). Anwendungsgebiete und -grenzen des Einsatzes von Atmosphärendruck-Mikroplasmen in der Hals-Nasen-Ohrenheilkunde, Teilvorhaben: Erprobung der therapeutischen Wirkung und Nebenwirkung des Plasmaprozesses am humanen Zellkultur- und Tiermodell, Akronym: Plasmaskop: Schlussbericht.

[184] Winter J., Nishime T.M.C., Glitsch S., Lühder H., Weltmann K.D. (2018). On the development of a deployable cold plasma endoscope. Contrib. Plasma Phys..

[185] Winter J., Nishime T.M.C., Bansemer R., Balazinski M., Wende K., Weltmann K.D. (2019). Enhanced atmospheric pressure plasma jet setup for endoscopic applications. J. Phys. D. Appl. Phys..

[186] Jablonowski H., Hoffmann U., Bansemer R., Bekeschus S., Gerling T., von Woedtke T. (2024). Characterization and comparability study of a series of miniaturized neon plasma jets. J. Phys. D. Appl. Phys..

[187] Jablonowski H., Rasnani K.H., Rataj R., do Nascimento F., Hoffmann U., Miebach L., Bansemer R., Bekeschus S., Weltmann K.D., von Woedtke T. (2025). Temporal analysis of a miniaturized plasma jet: A comprehensive study of the electrical and chemical characteristics. J. Phys. D. Appl. Phys..

